# K_2_PtCl_4_-induced structural reconstruction of high-entropy sulfide hollow nanospheres for enhanced hydrogen evolution

**DOI:** 10.1039/d6na00484a

**Published:** 2026-09-17

**Authors:** Yinghao Cui, Wenyang Zhang, Yusuke Yamauchi, Yoshiyuki Sugahara

**Affiliations:** a School of Advanced Science and Engineering, Waseda University 3-4-1 Okubo, Shinjuku Tokyo 169-8555 Japan ys6546@waseda.jp; b Kagami Memorial Research Institute for Materials Science and Technology, Waseda University 2-8-26 Nishiwaseda, Shinjuku Tokyo 169-0051 Japan; c Shenyang National Laboratory for Materials Science, Institute of Metal Research, Chinese Academy of Sciences 72 Wenhua Road Shenyang 110016 China wyzhang@imr.ac.cn; d School of Materials Science and Engineering, University of Science and Technology of China Hefei 230026 China; e Australian Institute for Bioengineering and Nanotechnology (AIBN), The University of Queensland Brisbane QLD 4072 Australia y.yamauchi@uq.edu.au; f Department of Materials Process Engineering, Graduate School of Engineering, Nagoya University Furo-cho, Chikusa-ku Nagoya 464-8603 Japan

## Abstract

We report the synthesis of K_2_PtCl_4_-treated high-entropy sulfide (HES) nanospheres *via* a two-step liquid-phase synthesis involving solvothermal formation of (Co,Ni,Cu,Zn,Mn)_3_S_4_ hollow spheres and subsequent K_2_PtCl_4_ solution treatment. The resulting nanospheres exhibit a well-defined hollow morphology. The K_2_PtCl_4_ solution treatment induces partial structural reconstruction of the HES framework, producing a Pt-containing amorphous region while retaining part of the crystalline spinel domain. The resulting catalyst also demonstrates excellent catalytic activity for the hydrogen evolution reaction (HER), delivering low overpotentials of 50 mV at 10 mA cm^−2^ in 1.0 M KOH and 49 mV at 10 mA cm^−2^ in 0.5 M H_2_SO_4_. It also demonstrates good long-term durability, retaining 71% and 98% of its initial current density after 60 h of continuous operation at constant potentials corresponding to an initial current density of 100 mA cm^−2^ under alkaline and acidic conditions, respectively, and maintaining almost constant polarization behavior after 5000 CV cycles under both conditions. These findings highlight the potential of K_2_PtCl_4_-treated HES as efficient and durable electrocatalysts for sustainable hydrogen production across a wide pH range.

## Abstract 2

K_2_PtCl_4_ solution treatment reconstructs the sulfide surface to generate Pt-containing amorphous regions, enabling efficient and durable HER in both acidic and alkaline electrolytes.

## Graphical abstract

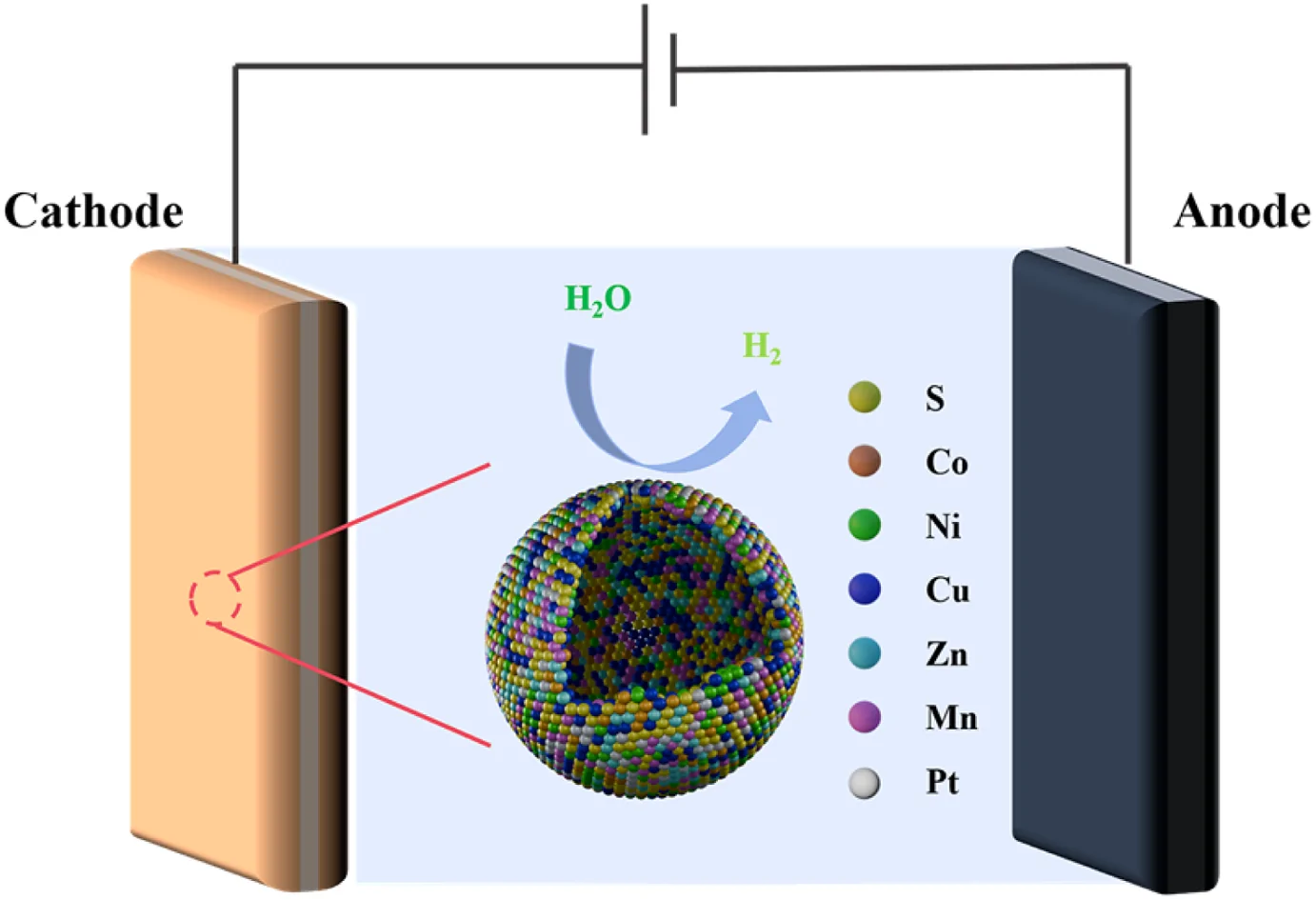

## 1. Introduction

Excessive reliance on fossil fuels has led to a global energy crisis and environmental degradation, highlighting the urgent need for alternative clean energy sources.^1–3^ Hydrogen is widely recognized as a promising energy carrier due to its high energy density and zero carbon emissions at the point of use, making it an ideal candidate for renewable energy storage and conversion.^4–7^ Among various hydrogen production strategies, electrochemical water splitting is considered an efficient approach for generating high-purity hydrogen, especially when powered by renewable energy sources.^8–10^ Although alkaline water electrolysis has gained significant attention due to its lower cost and superior durability compared to acidic electrolysis, the sluggish kinetics of the hydrogen evolution reaction (HER) in alkaline media remain a major challenge.^11^ This limitation results mainly from the additional water dissociation process involved in the alkaline Volmer step in the HER, which reduces its overall efficiency.^12^ The development of high-performance HER electrocatalysts with enhanced water dissociation and hydrogen adsorption capabilities is consequently crucial for advancing alkaline water electrolysis.

Pt-based materials are known to be the most efficient HER catalysts due to their near-optimal hydrogen-binding energy.^13,14^ Nevertheless, their catalytic performance is significantly lower in alkaline media than in acidic ones, primarily due to the water dissociation kinetics on Pt surfaces.^15,16^ Accordingly, efforts to facilitate water dissociation have been made to couple Pt with transition metal compounds.^17^ Transition metal oxides, sulfides, and hydroxides have been widely utilized as water dissociation promoters, accelerating the Volmer step and improving the overall HER efficiency.^18^ Pt single atoms coupled with Co/CoO heterostructures or supported on layered double hydroxides (LDHs) have been reported to promote alkaline HER.^17,19^ In addition to Pt-based compositional design, the construction of amorphous catalytic structures has also been recognized as an effective strategy for improving electrocatalytic activity.^20^ Their disordered coordination environments and abundant unsaturated sites can increase accessible active sites and facilitate adsorption/desorption processes during the HER.^20,21^ For example, Pt-modified amorphous systems, including Pt electrodeposited on amorphous MoS_*x*_ and amorphous MoO_*x*_-stabilized Pt species, have shown enhanced HER activity, highlighting the benefit of amorphous catalytic structures.^21,22^

High entropy sulfides (HES) have recently emerged as promising platforms for electrocatalysis due to their unique electronic structures, enhanced stability, and tunable active sites.^23–26^ The chemically diverse metal–sulfur framework of HES can provide a useful basis for surface reconstruction and the formation of partially amorphized multicomponent sulfide structures, while the retained sulfide framework can help maintain the overall morphology and structural stability.^27,28^ Inspired by recent discussion on the controllable synthesis of high-entropy materials, the fabrication of well-defined HES with tailored composition and distinct morphology remains an important objective.^29–31^ Establishing such a controllable synthetic strategy is therefore important for rational design of surface-modified HES-based electrocatalysts.

To address this issue, a two-step liquid-phase strategy is proposed in this work to convert pre-formed HES hollow nanospheres into K_2_PtCl_4_-treated HES with a Pt-doped amorphous region while retaining the hollow morphology and part of the crystalline domain. Through the two-step synthesis, the pre-formed HES was treated with a K_2_PtCl_4_ solution at 80 °C, resulting in Pt-associated reconstruction of the surface sulfide region while retaining the hollow morphology and the spinel phase. The resulting product was comprehensively characterized by X-ray diffraction (XRD), transmission electron microscopy (TEM), energy-dispersive X-ray spectroscopy (EDS), X-ray photoelectron spectroscopy (XPS), and inductively coupled plasma atomic emission spectroscopy (ICP), confirming the successful integration of Pt. The obtained material showed a surface-reconstructed structure with a partially amorphized surface region and a retained spinel crystalline domain. Pt-containing HES provides a general strategy to enhance both catalytic activity and durability by combining the multi-element synergy of high-entropy materials with incorporation of Pt, thus offering a promising pathway for the rational design of next-generation electrocatalysts for hydrogen evolution across diverse electrochemical environments.

## 2. Experimental section

### 2.1. Materials

Cobalt nitrate hexahydrate (Co(NO_3_)_2_·6H_2_O, 98%), nickel nitrate hexahydrate (Ni(NO_3_)_2_·6H_2_O, 98%), copper nitrate trihydrate (Cu(NO_3_)_2_·3H_2_O, 98%), zinc nitrate hexahydrate (Zn(NO_3_)_2_·6H_2_O, 98%), manganese nitrate hexahydrate (Mn(NO_3_)_2_·6H_2_O, 98%), potassium hydroxide (KOH, 99%), potassium tetrachloroplatinate(ii) (K_2_PtCl_4_), carbon disulfide (CS_2_, 99%), *N*,*N*,*N*′,*N*″,*N*″-pentamethyl diethylenetriamine (PMDTA, 98%), Nafion D521 solution (5%) and ethanol were all supplied by FUJIFILM Wako Pure Chemical. All the chemicals were used without further purification.

### 2.2. Synthesis of (Co,Ni,Cu,Zn,Mn)_3_S_4_

The experimental method used in this study was adapted from our previously reported approach.^32^ (Co,Ni,Cu,Zn,Mn)_3_S_4_ was synthesized by the solvothermal method. Specifically, 146 mg of Co(NO_3_)_2_·6H_2_O (0.5 mmol), 146 mg of Ni(NO_3_)_2_·6H_2_O (0.5 mmol), 121 mg of Cu(NO_3_)_2_·3H_2_O (0.5 mmol), 149 mg of Zn(NO_3_)_2_·6H_2_O (0.5 mmol), and 287 mg of Mn(NO_3_)_2_·6H_2_O (1 mmol) were dissolved in 10 ml of ethanol. After stirring for 30 min, 240 µl (4 mmol) of CS_2_ was added, followed by dropwise addition of 2 ml of PMDTA under continuous stirring. The resulting mixture was stirred for an additional 2 h. Subsequently, the reaction solution was transferred into a 50 ml Teflon-lined stainless-steel autoclave, which was sealed and heated at 180 °C for 12 h. After naturally cooling down to room temperature, the products were collected by centrifugation, washed with ethanol three times, and dried in a vacuum oven at 80 °C for 12 h.

### 2.3. Synthesis of K_2_PtCl_4_-treated HES

To obtain K_2_PtCl_4_-treated HES, 10 mg of K_2_PtCl_4_ (0.024 mmol) was first dissolved in 2 ml of deionized water at 80 °C to obtain a transparent solution. Subsequently, 25 mg of as-prepared (Co,Ni,Cu,Zn,Mn)_3_S_4_ powder was dispersed in the above solution through 10 min of ultrasonication to ensure uniform suspension. The mixture was then maintained under continuous stirring at 80 °C for 12 h. Finally, K_2_PtCl_4_-treated HES was obtained upon washing with ethanol and vacuum drying at 60 °C. To examine the effect of the acidic environment during K_2_PtCl_4_ solution treatment, an HNO_3_-treated HES control sample was prepared under the same pH conditions. Briefly, the pH of a K_2_PtCl_4_ aqueous solution was measured to be approximately 3.61, and the pH of an HNO_3_ aqueous solution was adjusted to the same value. Then, 25 mg of the as-prepared (Co,Ni,Cu,Zn,Mn)_3_S_4_ was dispersed in 2 ml of the pH-adjusted HNO_3_ aqueous solution and stirred at 80 °C for 12 h. The product was washed, followed by vacuum drying at 80 °C.

### 2.4. Synthesis of K_2_PtCl_4_-treated CoS, CoNiS, CoNiCuS, and CoNiCuZnS

A sequential approach was employed to synthesize various metal sulfides. The first step was adapted from our previously reported approach.^32^ To prepare unary CoS, 174 mg of Co(NO_3_)_2_·6H_2_O (0.6 mmol) was dissolved in 10 ml of ethanol, followed by 48 µl of CS_2_ (0.8 mmol) and 0.4 ml of PMDTA. For binary CoNiS, 174 mg of Co(NO_3_)_2_·6H_2_O (0.6 mmol) and 174 mg of Ni(NO_3_)_2_·6H_2_O (0.6 mmol) were dissolved in 10 ml of ethanol, with 96 µl of CS_2_ (1.6 mmol) and 0.8 ml of PMDTA added. Ternary CoNiCuS was synthesized by dissolving 174 mg of Co(NO_3_)_2_·6H_2_O (0.6 mmol), 174 mg of Ni(NO_3_)_2_·6H_2_O (0.6 mmol), and 145 mg of Cu(NO_3_)_2_·3H_2_O (0.6 mmol) in 10 ml of ethanol, and then adding 144 µl of CS_2_ (2.4 mmol) and 1.2 ml of PMDTA. Finally, quaternary CoNiCuZnS was synthesized by dissolving 174 mg of Co(NO_3_)_2_·6H_2_O (0.6 mmol), 174 mg of Ni(NO_3_)_2_·6H_2_O (0.6 mmol), 145 mg of Cu(NO_3_)_2_·3H_2_O (0.6 mmol), and 178 mg of Zn(NO_3_)_2_·6H_2_O (0.6 mmol) in 10 ml of ethanol, followed by the addition of 192 µl of CS_2_ (3.2 mmol) and 1.6 ml of PMDTA.

The solutions were then transferred to 50 ml Teflon-lined stainless-steel autoclaves to conduct solvothermal reactions at 180 °C for 12 h. The resulting products were washed with ethanol, followed by drying at 60 °C for 6 h. Twenty-five mg of each sample mentioned above were then dispersed in 2 ml of deionized water containing 10 mg of K_2_PtCl_4_ and stirred for 12 h at 80 °C. The final product was washed with deionized water and ethanol, followed by vacuum drying at 80 °C.

### 2.5. Material characterization

The obtained samples were characterized using the following techniques. X-ray diffraction (XRD, Rigaku SmartLab) with Fe Kα radiation (*λ* = 0.194 nm) was employed to analyze the crystalline phases. The microstructure and elemental distribution were examined using a transmission electron microscope (TEM, JEOL JEM-2100F) equipped with an energy-dispersive X-ray spectroscope (EDS) at an accelerating voltage of 200 kV. The surface morphology was observed using a field-emission scanning electron microscope (FESEM, Hitachi SU8240). The elemental composition was quantified by inductively coupled plasma atomic emission spectroscopy (ICP, Thermo Fisher iCAP Pro). Solid samples were fully dissolved in a mixed acid solution of HNO_3_ and HCl (1 : 3, v/v) at 250 °C, and the resulting clear solutions were subsequently diluted with deionized water. The valence states of elements in HES were investigated using X-ray photoelectron spectroscopy (XPS, JEOL JPS-9010MX). A BELSORP MINI II instrument was used to measure the nitrogen adsorption–desorption isotherms at 77 K. Sample pretreatment involved degassing under vacuum at 110 °C for 8 h. The specific surface areas were calculated by the Brunauer–Emmett–Teller (BET) method.

### 2.6. Electrochemical measurement

Electrochemical characterization was performed using a three-electrode single-compartment cell system with a BAS CHI 760EN electrochemical workstation under ambient conditions. All electrochemical characterization studies were conducted at room temperature in 1.0 M KOH and 0.5 M H_2_SO_4_ solutions. The working electrode was a 3 mm-diameter glassy carbon electrode loaded with the active material, while a carbon rod served as the counter electrode and a standard Hg/HgO electrode as the reference electrode. The catalyst ink was prepared by dispersing 4 mg of the sample in 1 ml of a 3 : 1 (v/v) ethanol–water mixture, followed by 20 min of sonication. Subsequently, 50 µl of a Nafion solution was added, and the mixture was sonicated for an additional 20 min to ensure homogeneity before coating onto the electrode. Then, 5 µl of the catalyst ink was dropped onto the glassy carbon electrode and dried, corresponding to a catalyst loading of approximately 0.27 mg cm^−2^. *iR* compensation was applied to all polarization curves to minimize the influence of solution resistance and enable more accurate evaluation of the electrocatalytic kinetics. Before the collection of polarization curves, the working electrode was electrochemically activated by performing several cyclic voltammetry (CV) scans until a stable voltammetric response was obtained. Linear sweep voltammetry (LSV) was conducted to assess the electrocatalytic activity of the synthesized catalysts for the HER, with all polarization curves measured at a scan rate of 5 mV s^−1^. All measured potentials were converted to reversible hydrogen electrode (RHE) values using the formula:*E* (*vs.* RHE) = *E* (*vs.* Hg/HgO) + 0.098 + 0.0592 × pH

Tafel plots for the catalysts were derived from the corresponding LSV curves. Electrochemical impedance spectroscopy (EIS) was performed in a frequency range of 0.1–100 000 Hz to analyze charge-transfer resistance (*R*_ct_) across the electrode/electrolyte interface. The working electrodes for the ECSA and durability measurements were prepared by depositing the catalyst inks onto carbon paper with an exposed geometric area of 1 cm^2^. Electrochemically active surface area (ECSA) was estimated based on the double-layer capacitance (*C*_dl_). The ECSA of a sample was estimated as ECSA = (*C*_dl_/*C*_s_) × *A*_geo_, where *C*_dl_ was determined by conducting cyclic voltammetry (CV) at different scan rates of 20, 40, 60, 80, and 100 mV s^−1^ in a non-faradaic potential region. The capacitive current density, calculated as Δ*j*/2 = (*j*_anodic_ − *j*_cathodic_)/2 at a selected potential, was plotted as a function of the scan rate, and *C*_dl_ was obtained from the slope of the linear fit. *C*_s_ is the specific capacitance of a flat surface, which was assumed to be 40 µF cm^−2^, and *A*_geo_ is the exposed geometric electrode area. Stability and durability were evaluated by chronoamperometry at a constant potential corresponding to an initial current density of 100 mA cm^−2^.

## 3. Results and discussion

### 3.1. Material characterization

K_2_PtCl_4_-treated HES was synthesized *via* a two-step strategy. (Co,Ni,Cu,Zn,Mn)_3_S_4_ hollow nanospheres were first prepared solvothermally using carbon disulfide as the sulfur source,^32^ with the hollow morphology attributable to the Kirkendall effect driven by different ion diffusion rates.^33^ In the subsequent step, the pre-formed (Co,Ni,Cu,Zn,Mn)_3_S_4_ was treated with a K_2_PtCl_4_ solution. To confirm the phase composition and crystallographic structure of the obtained material, XRD analysis was conducted first. As shown in Fig. 1, the diffraction patterns of the pristine sample can be assigned to a typical A_3_X_4_ spinel structure (*Fd*3̄*m*), supporting the formation of spinel-type (Co,Ni,Cu,Zn,Mn)_3_S_4_ (HES), consistent with our previous report.^32^ In the XRD pattern of K_2_PtCl_4_-treated HES, the overall diffraction profile still retains the spinel-related features, indicating that at least a part of the original spinel crystalline domain was preserved. However, the diffraction peaks became weaker and broader compared with those of pristine HES, suggesting a decrease in crystallinity after the K_2_PtCl_4_ solution treatment. Evolution of XRD patterns (Fig. S1) further shows that the diffraction peaks of HES gradually decreased in intensity with increasing K_2_PtCl_4_ solution treatment time, suggesting a gradual decrease in crystallinity during the reaction process. This decrease in crystallinity implies the possible formation of amorphous regions, which is further discussed based on the HRTEM observations below. Compared to pristine HES, no additional peaks are observed (Fig. 1), suggesting that no obvious crystalline Pt-containing impurity phase was formed. No additional crystalline impurity peaks were observed for the other control samples synthesized *via* the same method (Fig. S2 and S3).

**Fig. 1:**
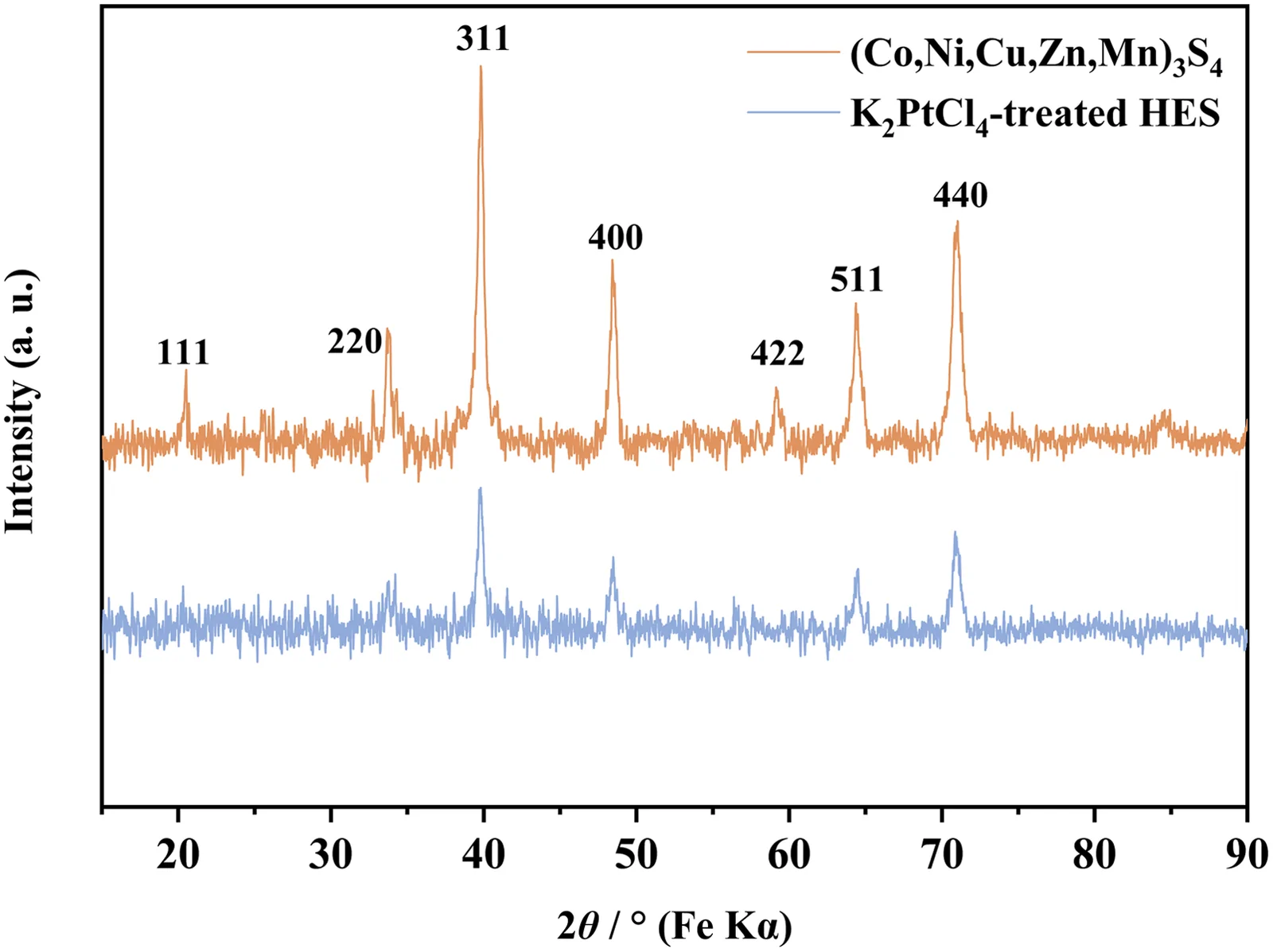
Powder XRD patterns of (Co,Ni,Cu,Zn,Mn)_3_S_4_ and K_2_PtCl_4_-treated HES.

The morphological and structural characteristics of K_2_PtCl_4_-treated HES were investigated using SEM and TEM techniques. As shown in Fig. 2a and b, both SEM and TEM images reveal a well-defined hollow spherical architecture, with the shell composed of interconnected nanograins. The high-resolution TEM image (Fig. 2c) displays clear lattice fringes with an interplanar spacing of 0.29 nm corresponding to the (311) plane of the spinel crystalline phase, which is close to that of the pristine HES sample (0.28 nm, Fig. S4), indicating that a part of the original spinel crystalline structure was retained after K_2_PtCl_4_ solution treatment. Amorphous regions are also observed, suggesting partial reconstruction of the surface region derived from the pre-formed HES framework. The corresponding SAED pattern (Fig. 2d) shows weak and diffuse diffraction rings, indicating reduced crystallinity after K_2_PtCl_4_ solution treatment. Compared with the clearer diffraction rings of pristine HES (Fig. S4), this result also demonstrates a decrease in crystallinity after the K_2_PtCl_4_ solution treatment. The remaining rings assigned to the (311) and (440) planes of the spinel structure suggest that a part of the original spinel crystalline domain is retained, while the diffuse background supports the formation of amorphous regions in K_2_PtCl_4_-treated HES. Considering the absence of clear XRD peak shifts and the mismatch between the ionic size of Pt^2+^ and those of the original metal cations in the spinel framework, lattice incorporation of Pt is unlikely.^34^ N_2_ adsorption–desorption measurements were also performed to compare the surface area before and after K_2_PtCl_4_ solution treatment. The specific surface area remained nearly unchanged after K_2_PtCl_4_ solution treatment (50 m^2^ g^−1^ → 48 m^2^ g^−1^), indicating that the porous structure and hollow morphology were essentially preserved (Fig. S5). Therefore, Pt species are more reasonably associated with the reconstructed surface sulfide region, as discussed below.

**Fig. 2:**
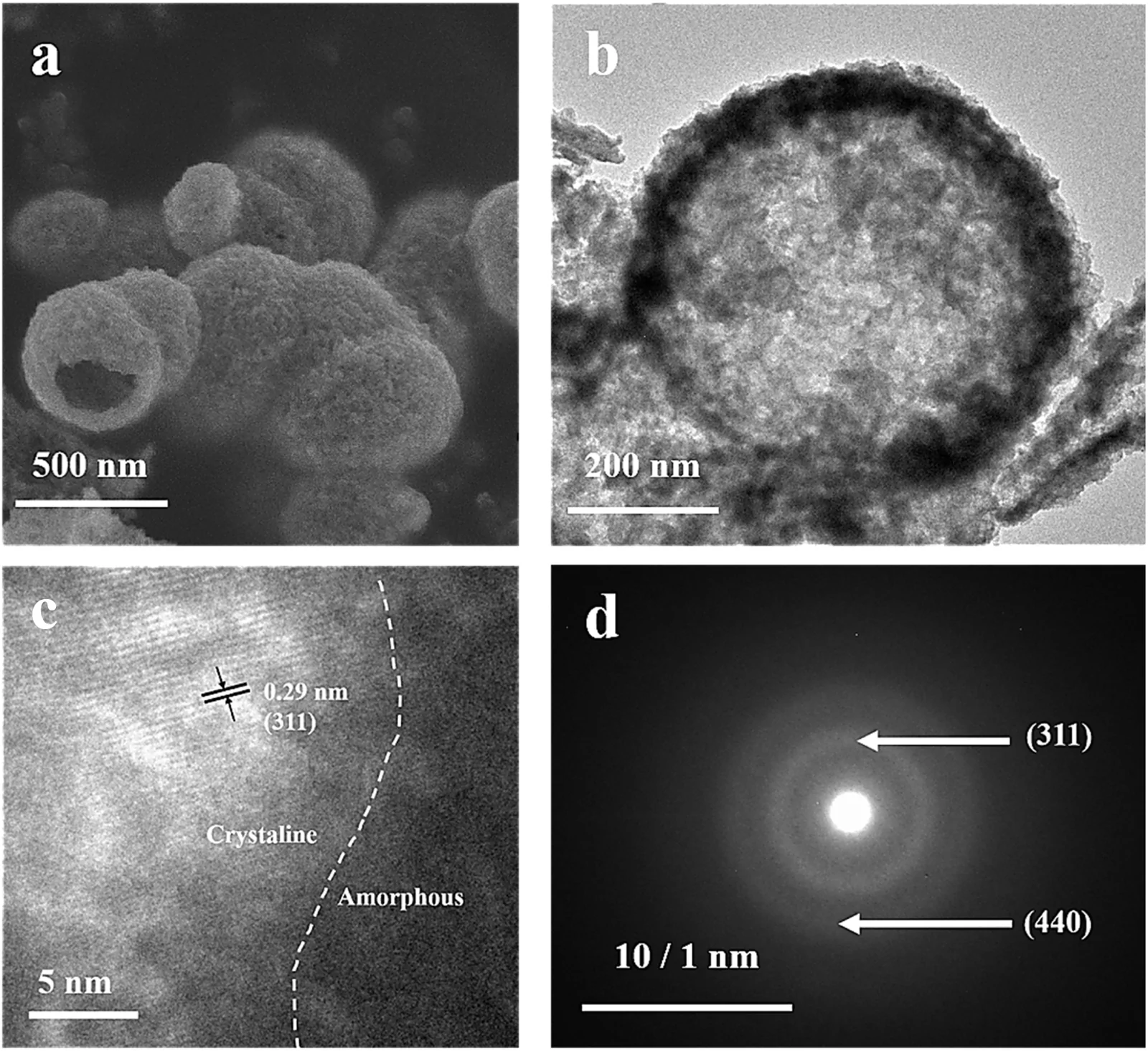
(a) SEM image of K_2_PtCl_4_-treated HES, (b) TEM image of K_2_PtCl_4_-treated HES, (c) HRTEM image of K_2_PtCl_4_-treated HES showing the crystal lattice and interface between the crystalline domain and amorphous region, and (d) SAED pattern of K_2_PtCl_4_-treated HES.

Based on the HRTEM and BET results, K_2_PtCl_4_-treated HES can be described as a surface-reconstructed structure derived from the pre-formed HES, in which the surface region is partially reconstructed while a part of the spinel crystalline framework is retained (Fig. 2c and S5). To clarify the origin of this structural feature, an acid-treatment control experiment was conducted. The K_2_PtCl_4_ solution used for Pt treatment showed a mildly acidic pH of 3.61, indicating that the liquid-phase treatment was performed under acidic conditions rather than in neutral ones. The XRD pattern of the pristine HES treated in pure water at 80 °C for 12 h shows that the spinel diffraction features were largely retained (Fig. S6). In contrast, the XRD pattern of the sample treated with HNO_3_ with the same pH as the K_2_PtCl_4_ solution shows a clear decrease in XRD peak intensities, suggesting that the acidic environment itself can reduce the crystallinity of the HES spinel framework. The TEM results of HNO_3_-treated HES further show the presence of the amorphous region, and the SAED pattern shows diffuse diffraction rings (Fig. S7), indicating a significant decrease in crystallinity after acid treatment. Therefore, the amorphous region observed in the K_2_PtCl_4_-treated sample is more reasonably associated with acid-induced surface reconstruction.^35–39^

TEM-EDS elemental mapping (Fig. 3) confirms the distribution of Co, Ni, Cu, Zn, Mn, S, and Pt throughout the entire shell, further suggesting successful integration of Pt. Notably, the Pt signal appears relatively stronger near the outer region of the hollow shell, further supporting that Pt introduction is closely associated with the surface/near-surface reconstruction process. To further examine the elemental distribution at a smaller length scale, high-magnification EDS elemental mapping was performed. As shown in Fig. S8, in the high-magnification EDS mapping, Co, Ni, Cu, Zn, Mn, and Pt remain distributed within the nanoparticles, and no obvious Pt-rich nanoparticles or isolated Pt-rich aggregates are observed. These observations suggest that the formation of discrete Pt nanoparticles can be reasonably excluded, and that the introduced Pt species were not present as obvious local aggregates within the HES matrix.

**Fig. 3:**
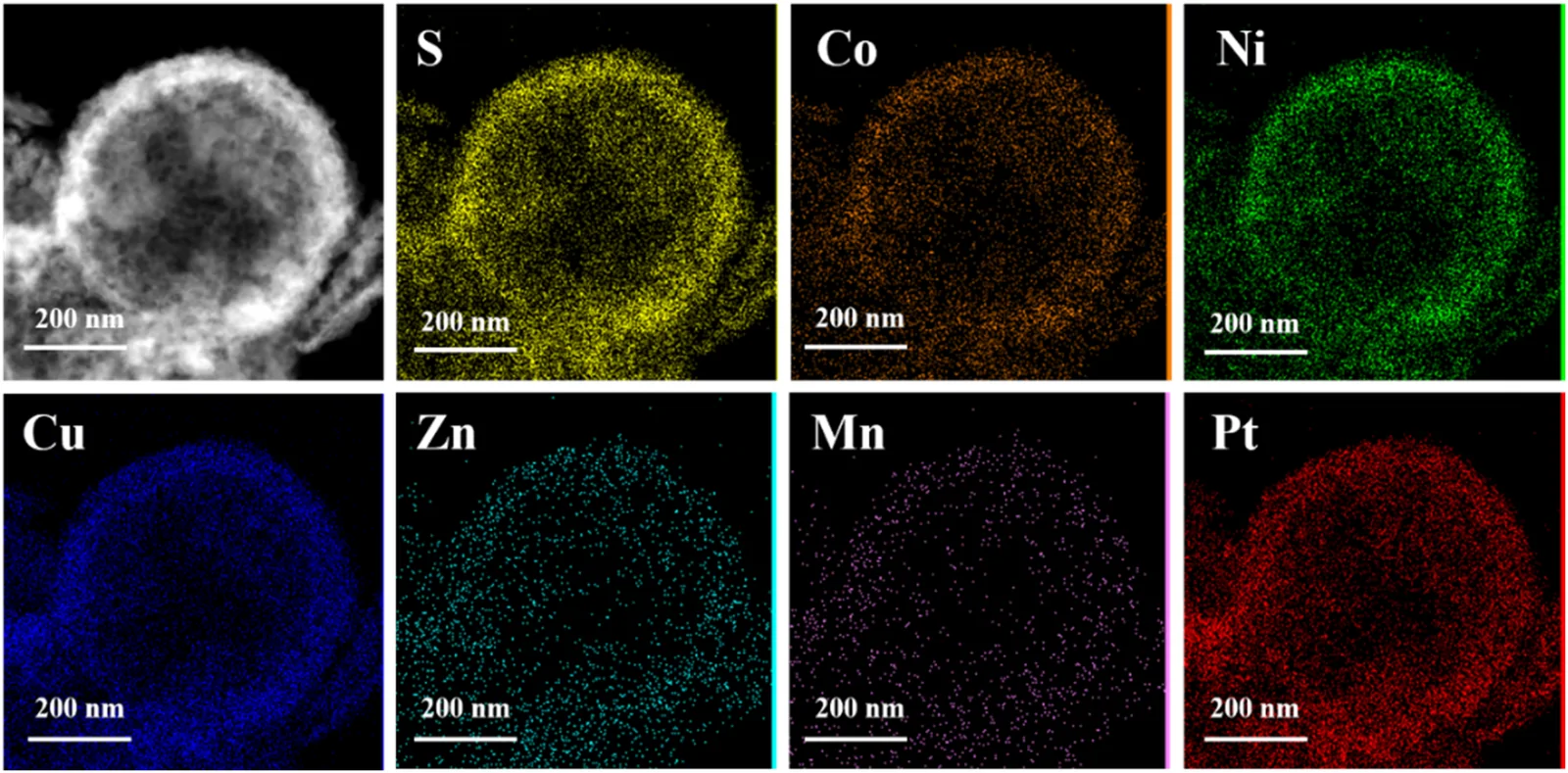
HAADF-STEM image and corresponding EDS elemental maps of S, Co, Ni, Cu, Zn, Mn, and Pt for K_2_PtCl_4_-treated HES.

The elemental contents of the pristine HES were determined by ICP, giving molar contents of Co = 0.0163 mmol, Ni = 0.0173 mmol, Cu = 0.0168 mmol, Zn = 0.0163 mmol, and Mn = 0.0215 mmol, and confirming the successful synthesis of the targeted high-entropy composition. After the K_2_PtCl_4_ solution treatment, the ICP results indicate the introduction of Pt and a resulting composition of Co = 0.0138 mmol, Ni = 0.0158 mmol, Cu = 0.0165 mmol, Zn = 0.0140 mmol, Mn = 0.0015 mmol, and the amount of Pt (0.0221 mmol, Table S1) supports the successful introduction of Pt. The incorporated Pt amount (0.0221 mmol) is comparable to the total decreased amount of host cations (0.0266 mmol), suggesting that Pt introduction is associated with partial surface dissolution during the K_2_PtCl_4_-induced reconstruction process rather than simple physical adsorption on the surface. For purposes of completeness, ICP analysis of the liquid phase, including the initial K_2_PtCl_4_ solution before Pt incorporation and the supernatant after centrifugation (after Pt incorporation), shows a similar trend (Table S2), in which the Pt concentration decreases by approximately 0.0220 mmol, an amount corresponding well to the total amount of dissolved host metals (0.0210 mmol), further supporting that Pt introduction is associated with surface reconstruction of HES.

The significantly reduced Mn content in the final product indicates that the elemental composition of the HES framework was changed during the K_2_PtCl_4_ solution treatment. This compositional change is likely associated with the reconstruction of the multi-component sulfide domain under acidic conditions. Previous studies have reported that Mn^2+^ exhibits coordination behavior different from that of Co^2+^ and Ni^2+^ analogues and has a weaker tendency to form sulfide precipitates than Zn^2+^, Ni^2+^, and Co^2+^, suggesting relatively weaker retention of Mn species under solution-based sulfide-forming conditions.^40,41^ These studies also indicate that sulfide precipitation can occur under acidic sulfide-containing conditions for metal ions with sufficiently small sulfide solubility products, whereas MnS is relatively soluble at low pH and Mn species can remain more soluble in solution at around pH 3.^40,41^ This tendency is also supported by the solubility products (*K*_sp_) of the corresponding metal sulfides. Previous studies have confirmed that the relative precipitation tendency of different metal sulfides is largely retained under acidic conditions, with MnS remaining more soluble than the other sulfides considered here.^40,42^ Therefore, the *K*_sp_ values are used here only as qualitative references to compare the relative solubility of the corresponding metal sulfides, rather than as direct quantitative parameters for the K_2_PtCl_4_-treatment process. Among the sulfides of Co, Ni, Cu, Zn, and Mn, manganese sulfide has a relatively larger *K*_sp_ value (MnS: 5.1 × 10^−15^, ZnS: 3.0 × 10^−25^, CoS: 5.9 × 10^−21^, NiS: 3.0 × 10^−21^, CuS: 7.9 × 10^−37^).^42–44^ This trend is generally consistent with the ICP results (Table S2), where Mn shows the largest decrease after K_2_PtCl_4_ solution treatment. Therefore, the lower Mn retention should be associated with the relatively higher solubility of Mn sulfide compared with the other metal sulfides. The Pt-related species were also considered from the perspective of sulfide stability. Although platinum hydroxylated species can exist in aqueous Pt–O–H systems, PtS has been reported to exhibit very low solubility in aqueous sulfide solutions (*K*_sp_ PtS: 9.9 × 10^−74^).^45^ Considering that the present treatment occurs on a sulfide-derived framework, Pt species are more reasonably considered to be stabilized in the reconstructed sulfide network as solid Pt–S-related species, rather than as a platinum hydroxide phase (*K*_sp_ Pt(OH)_2_: 10^−35^).^46^ The reduced Mn content is therefore considered as a compositional change accompanying the K_2_PtCl_4_-induced surface reconstruction process. Similar cases where Mn shows relatively lower incorporation compared with other metal species have also been reported in HES systems.^47^

Based on the characterization above, the K_2_PtCl_4_ solution treatment is considered to induce surface reconstruction of the pre-formed HES hollow nanospheres. The final product can therefore be described as a Pt-containing reconstructed sulfide-related amorphous surface formed on a spinel crystalline domain. Therefore, the following XPS analysis, as a surface-sensitive technique, mainly reflects the chemical environment of this reconstructed surface region rather than the retained inner spinel crystalline domain. Fig. 4 presents the XPS spectra of Co, Ni, Mn, Cu, Zn, S, and Pt in (Co,Ni,Cu,Zn,Mn)_3_S_4_ and K_2_PtCl_4_-treated HES. For pristine HES, the S 2p spectrum (Fig. 4a) shows two peaks at 161.9 and 163.0 eV, corresponding to sulfide S 2p_3/2_ and S 2p_1/2_, respectively. The 2p_3/2_ peak of Co consists of two peaks at 778.4 eV (Co^3+^) and 780.0 eV (Co^2+^), while the 2p_1/2_ peaks are located at 793.6 eV and 796.2 eV, respectively (Fig. 4b). In the case of Ni, the 2p_3/2_ spectrum reveals peaks at 853.0 eV (Ni^2+^) and 854.5 eV (Ni^3+^), and the 2p_1/2_ region shows peaks at 870.1 eV and 872.5 eV (Fig. 4c), which are consistent with reported work on other Ni-containing sulfide systems.^32,48^ The Cu 2p spectrum (Fig. 4d) exhibits Cu 2p_3/2_ and Cu 2p_1/2_ peaks at 932.1 eV and 952.0 eV, respectively, indicating the presence of Cu^2+^.^32^ The Zn 2p spectrum (Fig. 4e) shows Zn 2p_3/2_ and Zn 2p_1/2_ peaks at 1021.8 eV and 1044.8 eV, respectively, corresponding to Zn^2+^.^32,48^ The Mn 2p spectrum (Fig. 4f) shows two Mn 2p_3/2_ components at 640.9 eV and 643.0 eV, assigned to Mn^2+^ and Mn^3+^, respectively, and two Mn 2p_1/2_ components at 652.5 eV and 654.3 eV, corresponding to the same oxidation states.^26^ These results confirm the presence of multiple metal species in the pristine HES. The S 2p spectrum (Fig. 4a) of K_2_PtCl_4_-treated HES displays two characteristic peaks at 161.8 eV (2p_3/2_) and 163.0 eV (2p_1/2_), assigned to sulfide S^2−^ in the reconstructed surface region.^26^ In addition, a minor peak at 167.1 eV is attributed to S–O bonds, indicating partial surface oxidation of sulfur species. This could be related to the modified electronic environment induced by K_2_PtCl_4_-assisted Pt introduction and structural reconstruction, which may influence the oxidation tendency of surface sulfur. Compared with (Co,Ni,Cu,Zn,Mn)_3_S_4_, the S 2p spectrum of K_2_PtCl_4_-treated HES shifts slightly toward higher binding energy, suggesting that the K_2_PtCl_4_ solution treatment modifies the surface sulfur coordination environment and decreases the electron density around sulfur species.^26^ The transition metals 2p spectra also show clear changes after the K_2_PtCl_4_ solution treatment. Specifically, the Co 2p peaks shift toward lower binding energy, whereas the Ni 2p and Mn 2p peaks shift toward higher binding energy, while the Cu 2p and Zn 2p spectra exhibit relatively smaller changes (Fig. 4b–f). These element-dependent binding-energy shifts indicate that the K_2_PtCl_4_ solution treatment modifies the local electronic environments of the different metal sites. Similar asymmetric binding-energy shifts have been reported in multi-component systems, in which structural disorder and additional elemental incorporation lead to complex local electronic interactions among different metal sites.^26^ The Pt 4f spectrum (Fig. 4g) in K_2_PtCl_4_-treated HES features two primary peaks at 72.4 eV and 75.6 eV, corresponding to Pt 4f_7/2_ and 4f_5/2_, respectively,^49^ suggesting that Pt mainly exists as ionic Pt species rather than metallic Pt. The Pt 4f result suggests that ionic Pt species are associated with the reconstructed amorphous sulfide-derived surface region. Therefore, the XPS results indicate that the K_2_PtCl_4_ solution treatment changes the surface electronic structure of the multi-component sulfide-derived region containing ionic Pt species, rather than forming metallic Pt or simply preserving the original surface chemical environment.

**Fig. 4:**
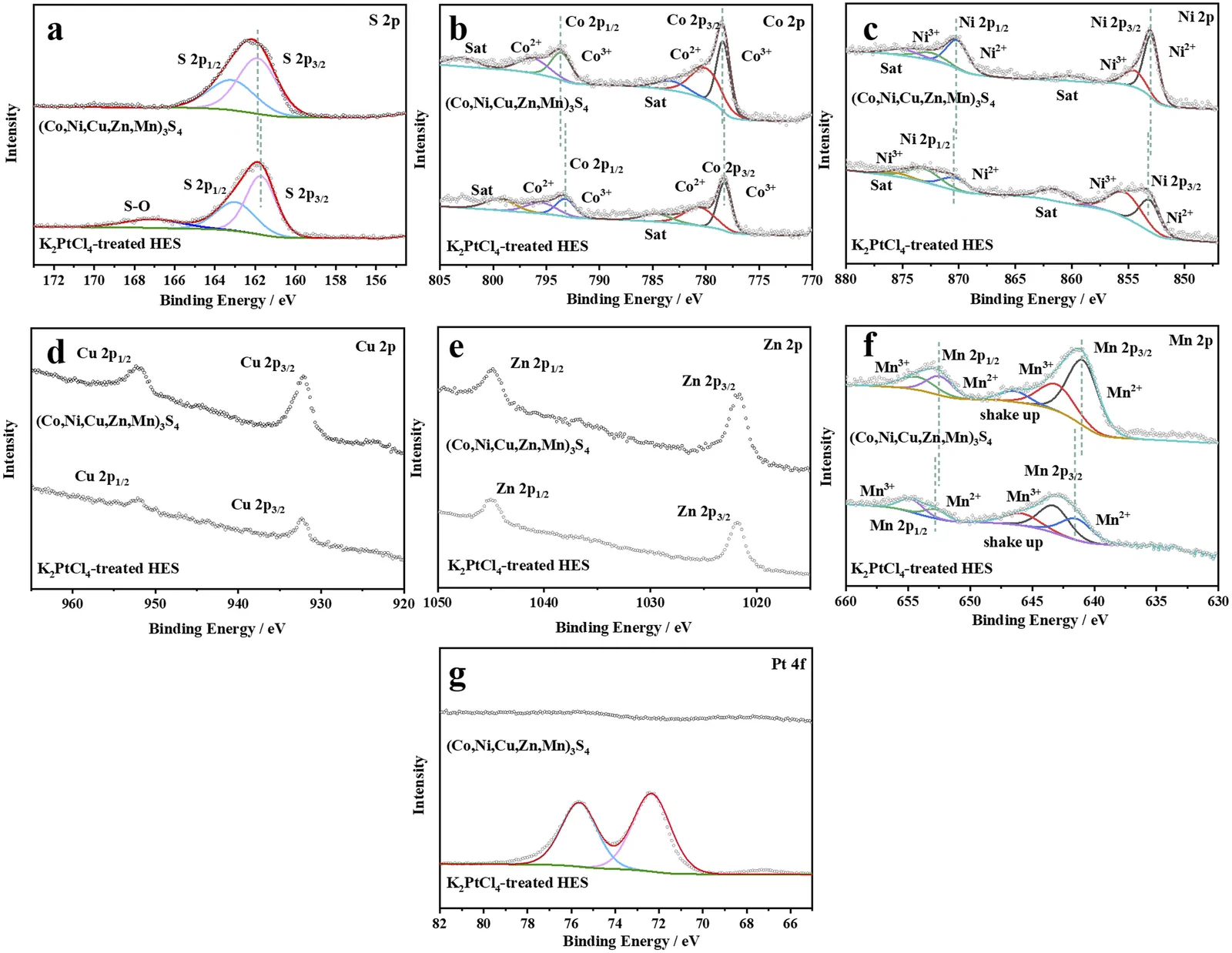
XPS spectra of K_2_PtCl_4_-treated HES for (a) S 2p, (b) Co 2p, (c) Ni 2p, (d) Cu 2p, (e) Zn 2p, (f) Mn 2p, and (g) Pt 4f.

### 3.2. Electrochemical characterization

The HER performances of the synthesized catalysts were evaluated using LSV measurements, as shown in Fig. 5a. By introducing Pt into systems with increasing compositional complexity (K_2_PtCl_4_-treated CoS → K_2_PtCl_4_-treated CoNiS → K_2_PtCl_4_-treated CoNiCuS → K_2_PtCl_4_-treated CoNiCuZnS → K_2_PtCl_4_-treated HES), the overpotentials at 10 mA cm^−2^ show a gradual decrease (Fig. 5b). This trend indicates that the HER activity is closely related to the K_2_PtCl_4_-induced reconstruction process. In particular, the formation of Pt-containing amorphous region is considered to provide highly active sites for the HER. Accordingly, K_2_PtCl_4_-treated HES achieves overpotentials of only 50 mV at 10 mA cm^−2^ and 213 mV at 200 mA cm^−2^, demonstrating excellent catalytic activity across a wide current density range. The pristine spinel HES before K_2_PtCl_4_ solution treatment exhibits much poorer HER activity (458 mV at 200 mA cm^−2^) than the K_2_PtCl_4_-treated HES, indicating that the original spinel phase alone is not sufficient to account for the high HER performance. Instead, the enhanced activity is mainly associated with the Pt-containing reconstructed surface sulfide region formed during K_2_PtCl_4_ solution treatment, while the retained spinel framework may help maintain structural continuity and charge transfer pathways. Tafel slope analysis (Fig. 5c) further supports the improved reaction kinetics as the compositional complexity increases. The Tafel slope of (Co,Ni,Cu,Zn,Mn)_3_S_4_ is calculated to be 161.1 mV dec^−1^, indicating relatively slow HER kinetics, while K_2_PtCl_4_ solution treatment and the formation of Pt-containing amorphous region led to a significant decrease in the Tafel slope to 55.0 mV dec^−1^, suggesting enhanced catalytic kinetics. In contrast to recently reported high-entropy catalysts, transition metal sulfides, and Pt-based systems (Table S3), the present catalyst exhibits a competitive combination of overpotential and Tafel slope. The control groups (K_2_PtCl_4_-treated CoS, K_2_PtCl_4_-treated CoNiS, K_2_PtCl_4_-treated CoNiCuS, and K_2_PtCl_4_-treated CoNiCuZnS) exhibit Tafel slopes ranging from 111.0 to 70.3 mV dec^−1^, following a similar trend to their overpotential results. These observations suggest that the enhanced HER kinetics of K_2_PtCl_4_-treated HES are mainly associated with Pt-containing amorphous regions generated during the reconstruction process. This behavior is consistent with the structural analysis, where K_2_PtCl_4_ solution treatment generates a Pt-containing amorphous sulfide region; such effects are generally associated with modified hydrogen adsorption behavior and an increased number of accessible active sites in high-entropy systems.^17,50–52^ Although changes in surface area can influence electrocatalytic performance, the BET surface areas of the samples show only limited variation after K_2_PtCl_4_ solution treatment (Fig. S5), suggesting that the performance enhancement cannot be solely attributed to surface area effects. To further evaluate the contribution of electrochemically accessible surface area, ECSA was estimated based on the *C*_dl_, as shown in Fig. S9 and S10. K_2_PtCl_4_-treated HES exhibits the highest *C*_dl_ value of 9.80 mF cm^−2^, corresponding to an ECSA of 245 cm^2^, exceeding those of pristine HES and the K_2_PtCl_4_-treated CoS, CoNiS, CoNiCuS, and CoNiCuZnS controls (82, 74, 121, 143, and 128 cm^2^). The ECSA results support the contribution of electrochemically accessible surface area to the HER performance, as K_2_PtCl_4_-treated HES shows the largest ECSA among the samples. This indicates that K_2_PtCl_4_-induced reconstruction of the HES surface increases the electrochemically accessible surface area, thereby providing more exposed active sites for the reaction. Based on the structural characterization, this enhanced HER performance is more reasonably associated with the reconstructed amorphous surface region formed during K_2_PtCl_4_ solution treatment, together with Pt introduction, enlarged ECSA, and retained spinel crystalline domain.^47,48,53^ The retained crystalline domain may further contribute to structural continuity and charge transfer. EIS measurements were conducted to further evaluate the charge transfer properties of the catalysts, and the corresponding Nyquist plots were fitted using an equivalent circuit model. As shown in Fig. 5d, K_2_PtCl_4_-treated HES exhibits the lowest *R*_ct_ among all samples, followed by the K_2_PtCl_4_-treated CoNiCuZnS, K_2_PtCl_4_-treated CoNiCuS, K_2_PtCl_4_-treated CoNiS, and K_2_PtCl_4_-treated CoS control samples, whereas pristine HES shows the highest *R*_ct_. The significantly reduced *R*_ct_ of HES suggests a more efficient charge transfer across the electrode/electrolyte interface, thereby facilitating faster HER kinetics.

**Fig. 5:**
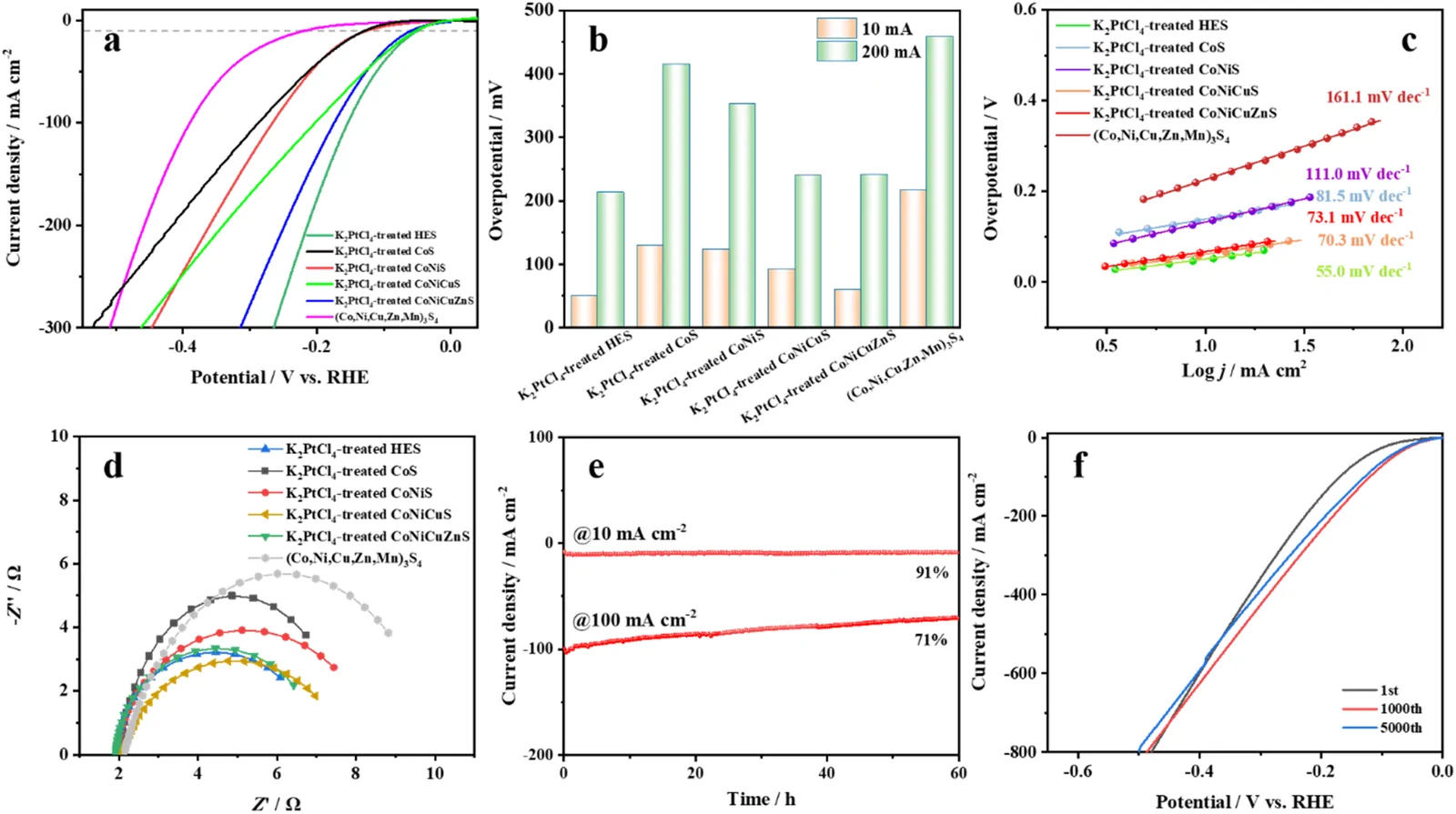
Alkaline HER performances of K_2_PtCl_4_-treated HES. (a) Linear sweep voltammetry (LSV) curves of different catalysts in 1.0 M KOH, (b) overpotentials at 10 and 200 mA cm^−2^ derived from LSV data, (c) Tafel plots of all samples, (d) Nyquist plots of different samples, (e) 60 h stability of K_2_PtCl_4_-treated HES in an alkaline environment, and (f) LSV curves after the 1st, 1000th, 5000th CV cycles.

The long-term stability of the catalysts was evaluated using chronoamperometry (*i*–*t*) measurements at constant potentials, as shown in Fig. 5e. K_2_PtCl_4_-treated HES demonstrated exceptional durability, maintaining a relatively stable current density over the 60 h testing period at a potential corresponding to an initial current density of 100 mA cm^−2^. To further evaluate the structural stability of the catalyst, post-stability characterization studies were conducted. The SEM image of K_2_PtCl_4_-treated HES after the *i*–*t* test shows that the overall morphology of the hollow nanospheres is largely preserved (Fig. S11), indicating that no significant structural collapse or aggregation occurs during prolonged electrolysis. XRD analysis conducted after the 60 h *i*–*t* stability tests in 1 M KOH shows that the diffraction peaks characteristic to the spinel structure are largely retained after the stability test (Fig. S12), indicating that the crystalline domain is preserved under operating conditions. The enhanced stability is likely related to the retained spinel framework in the surface-reconstructed HES structure, which helps maintain the hollow nanosphere architecture, reconstructed surface region, and corresponding surface active sites during prolonged electrolysis.^54,55^ In Fig. 5f, LSV curves recorded after the 1st, 1000th, and 5000th CV cycles show that the overpotential at 200 mA cm^−2^ decreases by 33 mV from the 1000th to the 5000th cycle (226 mV → 193 mV), indicating an enhancement of catalytic performance. To investigate the origin of the observed decrease in overpotential after cycling, ECSA was estimated from the *C*_dl_ before and after the activation process, as shown in Fig. S13. The results show that the *C*_dl_ value increases after cycling (4.02 → 9.80 mF cm^−2^), corresponding to an increase in the estimated ECSA (101 cm^2^ → 245 cm^2^), which indicates an increase in electrochemically accessible surface area. This suggests that additional active sites become exposed during the electrochemical process, which may contribute to the enhanced catalytic performance. The activation may be associated with the gradual exposure or further reconstruction of the Pt-doped amorphous HES region under electrochemical conditions, resulting in an increased number of accessible active sites for the HER.^56,57^ The retained spinel framework may help maintain structural continuity during long-term cycling, while the reconstructed surface region remains accessible for the HER.^53^ This result underscores the dynamic nature and robustness of Pt-containing HES in alkaline HER environments.

The HER performance of the catalysts was further evaluated under acidic conditions (0.5 M H_2_SO_4_) to investigate their behavior across different electrolytic environments. As shown in Fig. 6a, a similar trend to that observed under alkaline conditions was observed, with the overpotential at 10 mA cm^−2^ decreasing gradually from K_2_PtCl_4_-treated CoS to K_2_PtCl_4_-treated HES. Notably, the K_2_PtCl_4_-treated HES exhibited overpotentials of only 49 mV at 10 mA cm^−2^ and 223 mV at 200 mA cm^−2^, indicating superior catalytic activity in acidic media (Fig. 6b). Tafel slope analysis under acidic conditions (Fig. 6c) further confirms the enhanced HER kinetics. K_2_PtCl_4_-treated HES displays a Tafel slope of 51.4 mV dec^−1^, indicating accelerated HER kinetics and more efficient overall charge-transfer processes. Compared with the values under alkaline conditions, the lower overall Tafel slopes under acidic conditions are attributable to the proton-rich environment and the favorable electronic configuration of the catalyst, which together promote faster hydrogen evolution kinetics.^15,16^ EIS reveals a significantly reduced charge transfer resistance for K_2_PtCl_4_-treated HES (Fig. 6d), further validating its superior interfacial charge transfer kinetics in acidic medium. Consistent with the alkaline results, the ECSA estimated from *C*_dl_ also increases with compositional complexity under acidic conditions (Fig. S14 and S15), suggesting that the increased number of electrochemically accessible sites contributes to the acidic HER performance. The *C*_dl_ values of pristine HES, K_2_PtCl_4_-treated CoS, CoNiS, CoNiCuS, CoNiCuZnS, and HES are 4.11, 3.50, 5.92, 6.05, 6.48, and 11.97 mF cm^−2^, respectively, corresponding to ECSA values of 103, 88, 148, 151, 162 and 300 cm^2^, respectively. The long-term operational durability is shown in Fig. 6e, where the catalyst maintains a relatively stable current density over a 60 h *i*–*t* test at a constant potential corresponding to an initial current density of 100 mA cm^−2^, demonstrating excellent electrochemical stability. The superior activity of K_2_PtCl_4_-treated HES further reflects the favorable role of the Pt-modified multi-component sulfide domain. SEM and XRD analyses conducted after the 60 h *i*–*t* stability test in 0.5 M H_2_SO_4_ show that the hollow morphology and the diffraction peaks characteristic to the spinel phase are largely preserved after acidic electrolysis (Fig. S11 and S12), confirming its structural robustness under acidic conditions. Additionally, the LSV curves obtained after the 1st, 1000th, and 5000th CV cycles (Fig. 6f) show a clear activation trend. The overpotential at 200 mA cm^−2^ decreases by 61 mV after 1000 cycles compared to the initial scan (308 mV → 247 mV). After 5000 cycles, the overpotential was 268 mV, remaining 40 mV lower than the initial value, although it increased by 21 mV relative to that after 1000 cycles. The increased *C*_dl_ after 5000 cycles (4.02 mF cm^−2^ → 11.97 mF cm^−2^) (Fig. S16) corresponds to an increase in the estimated ECSA (101 cm^2^ → 300 cm^2^), suggesting improved electrochemical accessibility of the catalyst surface during cycling. Such trends are consistent with those observed under alkaline conditions, confirming the structural robustness and adaptive catalytic nature of K_2_PtCl_4_-treated HES in both acidic and alkaline environments. These findings confirm the excellent HER activity of K_2_PtCl_4_-treated HES across a wide pH range.

**Fig. 6:**
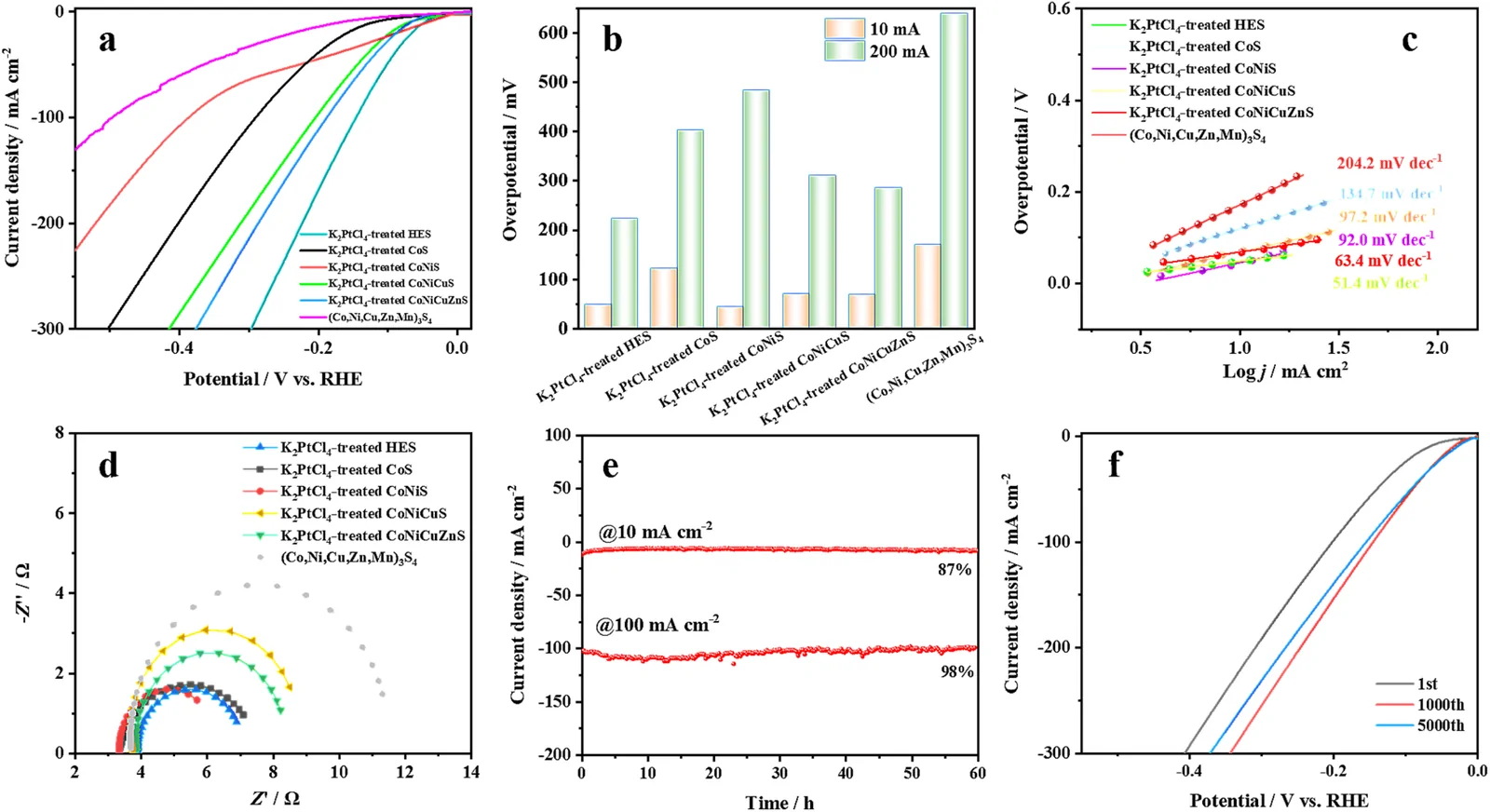
Acidic HER performances of K_2_PtCl_4_-treated HES. (a) Linear sweep voltammetry (LSV) curves of different catalysts in 0.5 M H_2_SO_4_, (b) overpotentials at 10 and 200 mA cm^−2^ derived from LSV data, (c) Tafel plots of all samples, (d) Nyquist plots of different samples, (e) 60 h stability of K_2_PtCl_4_-treated HES in an acidic environment, and (f) LSV curves after the 1st, 1000th, 5000th CV cycles.

## 4. Conclusions

A highly efficient electrocatalyst for the HER was synthesized through a two-step liquid-phase synthesis involving solvothermal formation of (Co,Ni,Cu,Zn,Mn)_3_S_4_ hollow spheres and subsequent K_2_PtCl_4_ solution treatment. The K_2_PtCl_4_ treatment induced surface reconstruction of the HES domain, forming a Pt-containing reconstructed amorphous surface region while retaining the spinel domain. K_2_PtCl_4_-treated HES with the reconstructed amorphous sulfide region shows excellent HER performance, achieving low overpotentials of 213 mV and 223 mV at 200 mA cm^−2^ in 1.0 M KOH and 0.5 M H_2_SO_4_, respectively. Furthermore, it demonstrates excellent long-term durability, retaining 71% and 98% of its initial density after 60 h of continuous operation at constant potentials corresponding to an initial current density of 100 mA cm^−2^ under alkaline and acidic conditions, respectively, and maintaining almost constant polarization behavior after 5000 CV cycles. These findings highlight the potential of K_2_PtCl_4_-treated HES with a Pt-doped amorphous sulfide-related region as an efficient and durable electrocatalyst for hydrogen production across a wide pH range.

## Author contributions

Yinghao Cui: conceptualization, methodology, formal analysis, investigation, data curation, writing – original draft. Wenyang Zhang: methodology, validation, writing – review & editing, supervision. Yusuke Yamauchi: writing – review & editing, supervision, funding acquisition. Yoshiyuki Sugahara: writing – review & editing, supervision.

## Conflicts of interest

The authors declare that they have no known competing financial interests or personal relationships that could have appeared to influence the work reported in this paper.

## Supplementary Material

NA-OLF-D6NA00484A-s001

## Data Availability

The data supporting this article have been included as part of the supplementary information (SI) file. Supplementary information: additional XRD patterns, electron microscopy images and elemental maps, nitrogen adsorption–desorption isotherms, ICP elemental analyses, electrochemical characterization and stability data, and a comparison of HER performance with selected literature reports. Ref. 58–68 are cited in the SI. See DOI: https://doi.org/10.1039/d6na00484a.
