# Supplementary material for: K2PtCl4-induced structural reconstruction of high-entropy sulfide hollow nanospheres for enhanced hydrogen evolution
Source: Nanoscale Adv. 2026 Sep 17. Online ahead of print. doi: 10.1039/d6na00484a (PMC13687670; doi:10.1039/d6na00484a)
Supplement: Supplementary file 1 [file NA-OLF-D6NA00484A-s001.pdf]

Supplementary Information (SI) for Nanoscale Advances.
This journal is © The Royal Society of Chemistry

Supplementary Information

K_2PtCl_4-Induced Structural Reconstruction of High-Entropy Sulfide Hollow Nanospheres for Enhanced Hydrogen Evolution

Yinghao Cui [a,b], Wenyang Zhang [c, d] *, Yusuke Yamauchi [e, f,] *, Yoshiyuki Sugahara [a, b,] *

a *School of Advanced Science and Engineering, Waseda University, 3-4-1 Okubo, Shinjuku, Tokyo 169-8555, Japan*
b *Kagami Memorial Research Institute for Materials Science and Technology, Waseda University, 2-8-26 Nishiwaseda, Shinjuku, Tokyo 169-0051, Japan*
c *Shenyang National Laboratory for Materials Science, Institute of Metal Research, Chinese Academy of Sciences, 72 Wenhua Road, Shenyang 110016, China*
d *School of Materials Science and Engineering, University of Science and Technology of China, Hefei 230026, China*
e *School of Chemical Engineering and Australian Institute for Bioengineering and Nanotechnology (AIBN), The University of Queensland, Brisbane, QLD 4072, Australia*
f *Department of Materials Process Engineering, Graduate School of Engineering, Nagoya University, Furo-cho, Chikusa-ku, Nagoya 464-8603, Japan*

*Corresponding author email: Wenyang Zhang: wyzhang@imr.ac.cn
Yusuke Yamauchi: y.yamauchi@uq.edu.au
Yoshiyuki Sugahara: ys6546@waseda.jp

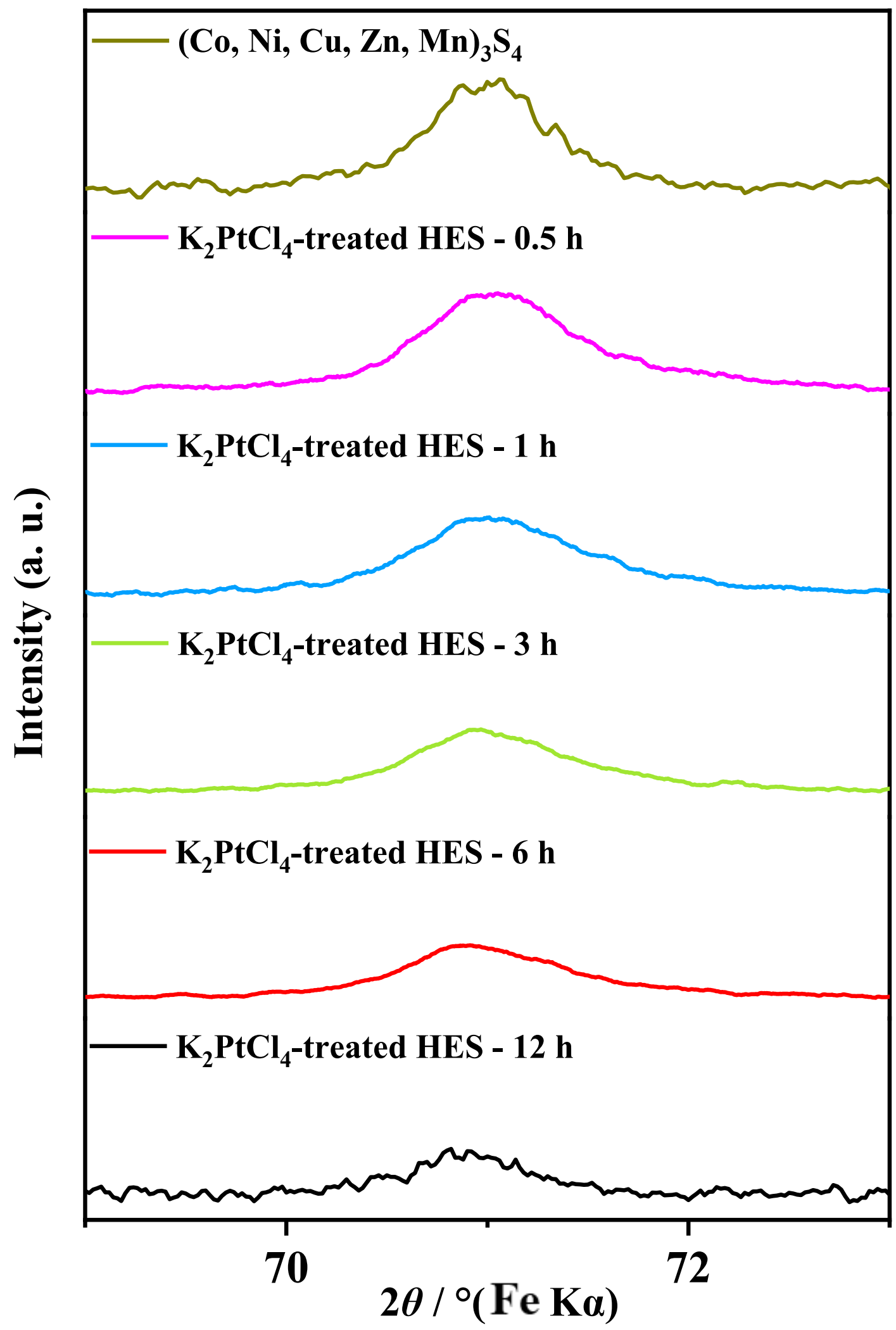

Fig. S1 XRD patterns of K_2PtCl_4-treated HES with different treatment lengths..

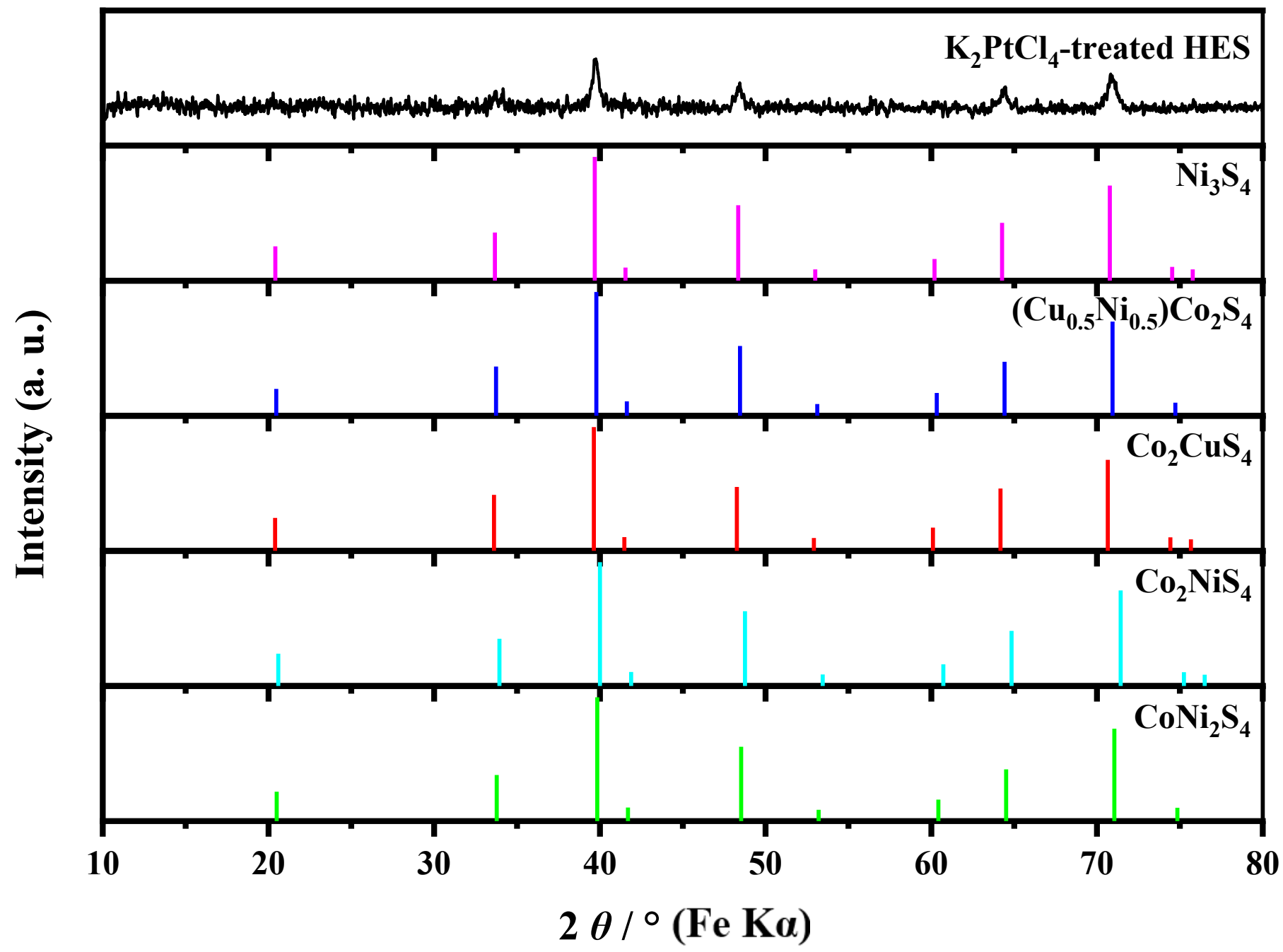

Fig. S2 Comparison of XRD pattern of K_2PtCl_4-treated HES with other A_3X_4-type spinel sulfides: $CoNi_2S_4$, Co_2NiS_4, Co_2CuS_4, $(Cu_{0.5}Ni_{0.5})Co_2S_4$, and Ni_3S_4.

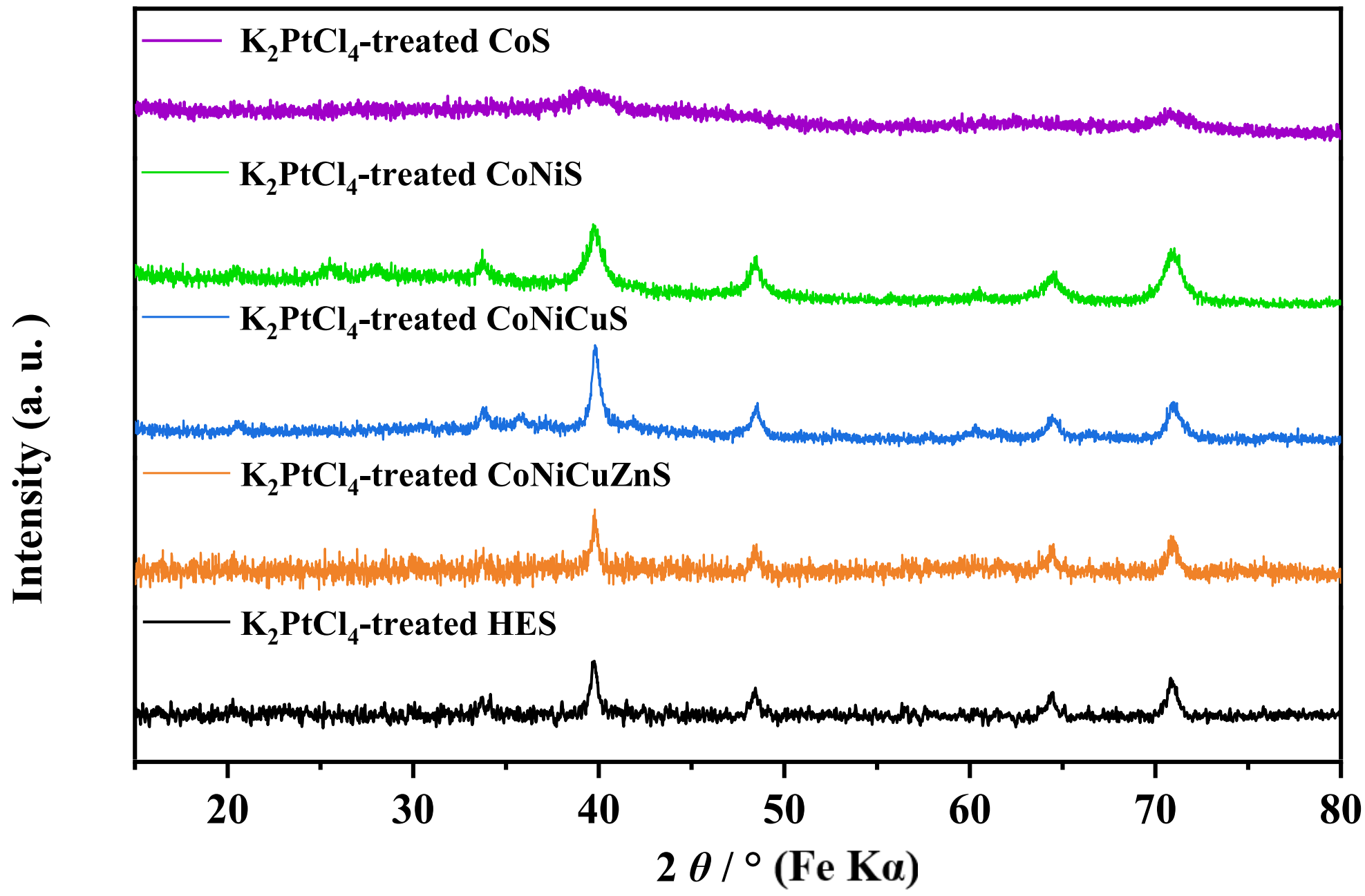

Fig. S3 XRD patterns of K_2PtCl_4-treated CoS, K_2PtCl_4-treated CoNiS, K_2PtCl_4-treated CoNiCuS, K_2PtCl_4-treated CoNiCuZnS andK_2PtCl_4-treated HES.

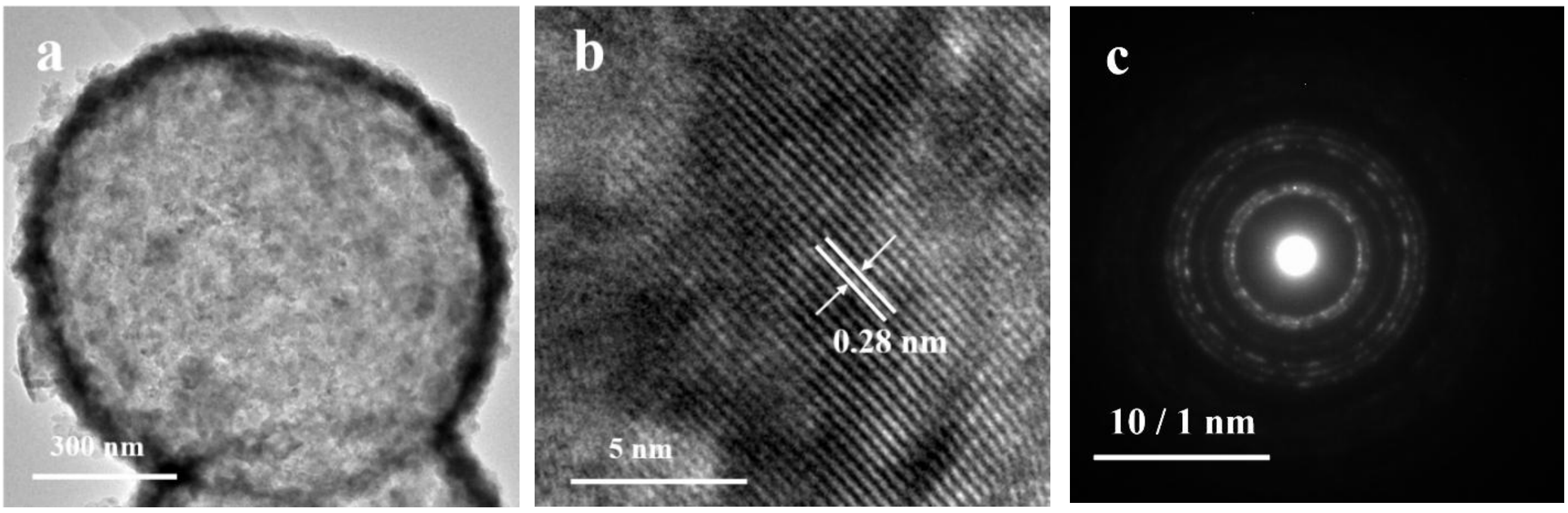

Fig. S4 (a) TEM image of $(Co,Ni,Cu,Zn,Mn)_3S_4$, (b) HRTEM image of $(Co,Ni,Cu,Zn,Mn)_3S_4$ with crystal lattice, and (c) SAED pattern.

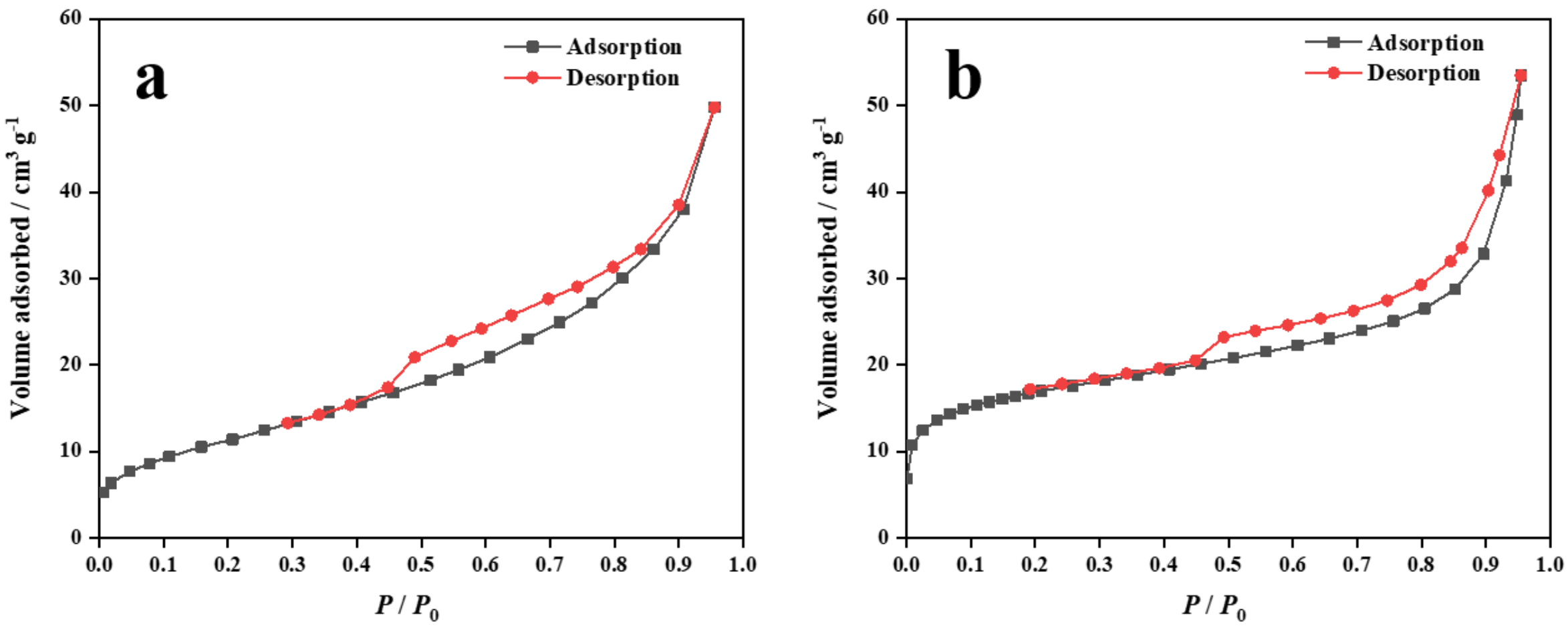

Fig. S5 N_2 adsorption-desorption isotherms of (a) $(Co,Ni,Cu,Zn,Mn)_3S_4$ and (b) K_2PtCl_4-treated HES.

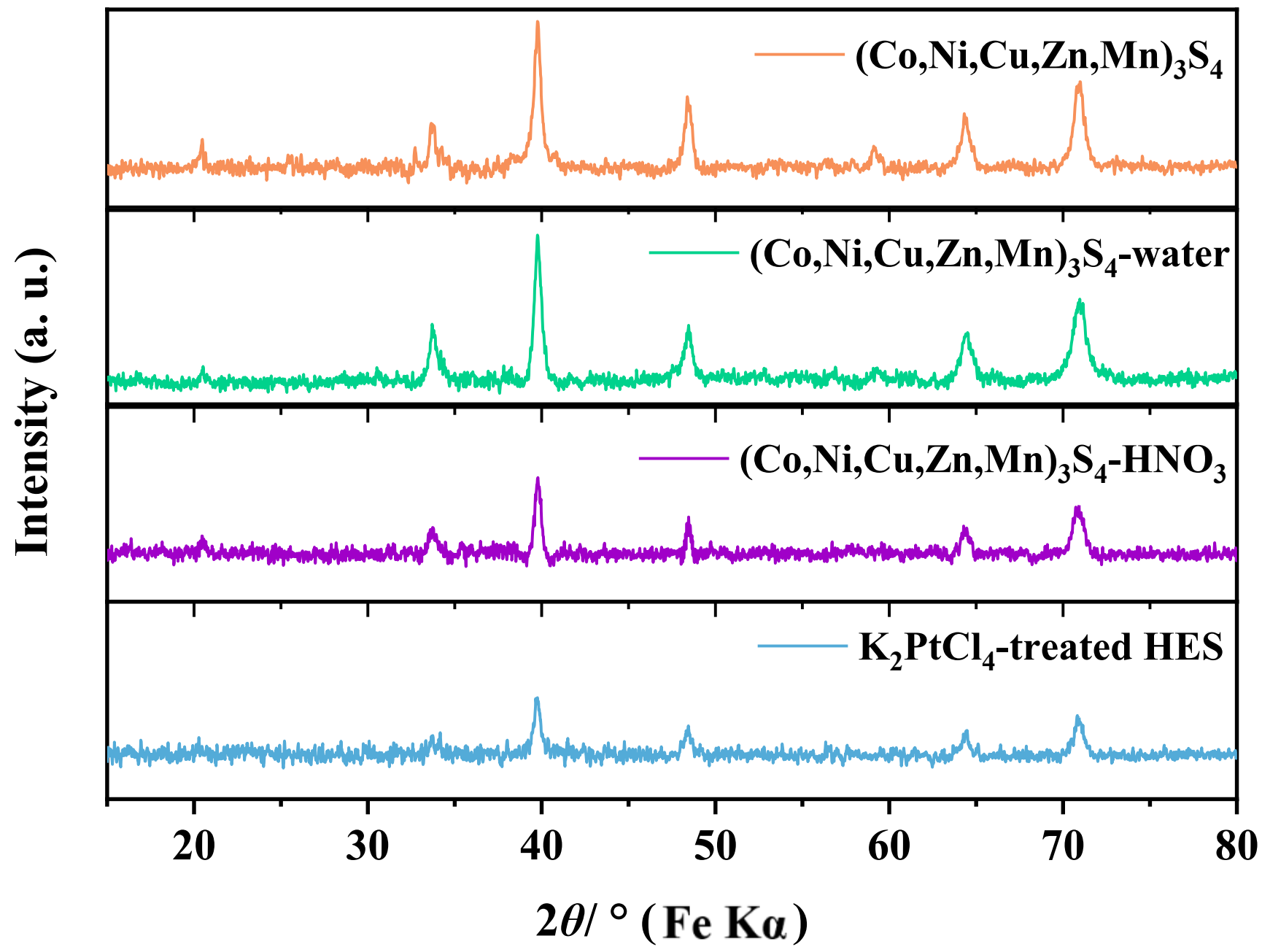

Fig. S6 XRD patterns of (Co,Ni,Cu,Zn,Mn)$_3$S$_4$, (Co,Ni,Cu,Zn,Mn)$_3$S$_4$ treated with water and HNO$_3$, and K$_2$PtCl$_4$-treated HES.

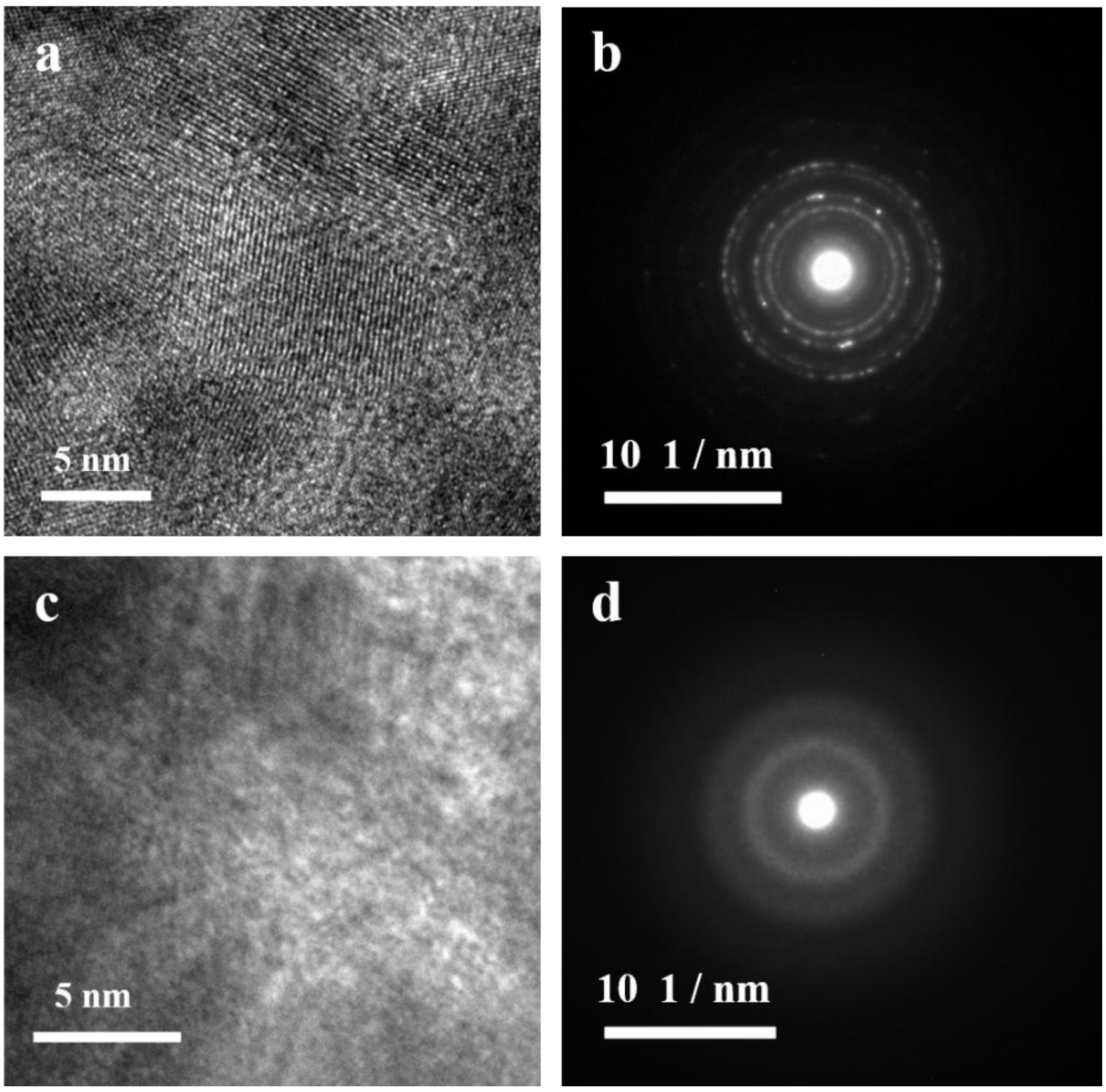

Fig. S7 TEM images of the pH-adjusted HNO_3-treated $(Co,Ni,Cu,Zn,Mn)_3S_4$ sample. (a) TEM image of a crystalline region and (b) the corresponding SAED pattern before treatment; (c) TEM image of amorphous region and (d) the corresponding SAED pattern after treatment.

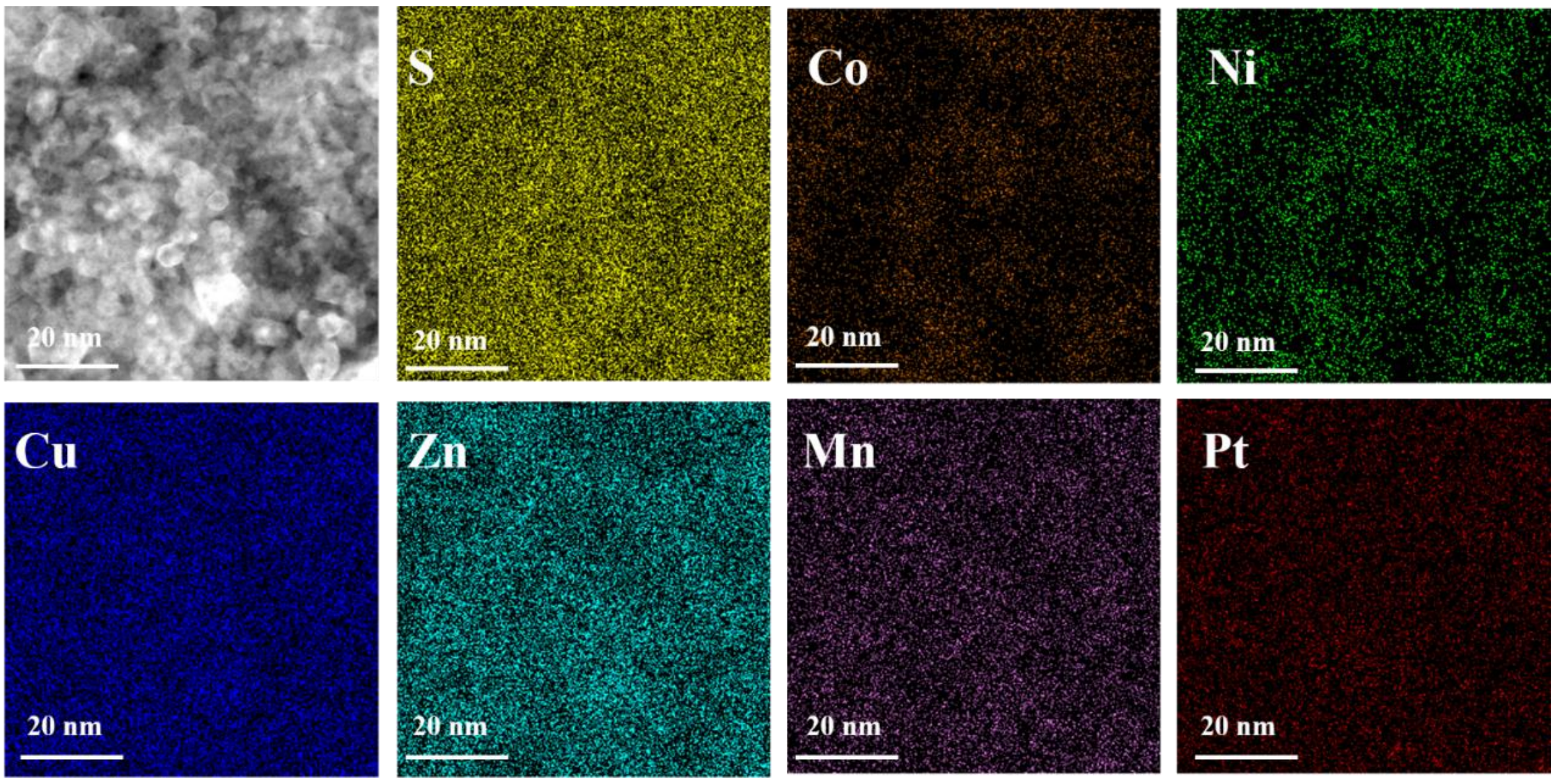

Fig. S8 High-magnification HAADF-STEM image and corresponding EDS elemental maps of S, Co, Ni, Cu, Zn, Mn, and Pt of K_2PtCl_4-treated HES.

Table S1. Metal contents of (Co, Ni, Cu, Zn, Mn)$_3$S$_4$ and K$_2$PtCl$_4$-treated HES.

Sample	Metal content/mmol					
	**Co**	**Ni**	**Cu**	**Zn**	**Mn**	**Pt**
(Co, Ni, Cu, Zn, Mn)$_3$S$_4$	0.0163	0.0173	0.0168	0.0163	0.0215	0
K$_2$PtCl$_4$-treated HES	0.0138	0.0158	0.0165	0.0140	0.0015	0.0221

Table S2. Metal contents of the initial K$_2$PtCl$_4$ solution and the supernatant after Pt incorporation.

Sample	Metal content/mmol					
	**Co**	**Ni**	**Cu**	**Zn**	**Mn**	**Pt**
K$_2$PtCl$_4$ Solution	0	0	0	0	0	0.0230
Supernatant after reaction	0.0013	0.0005	0.0003	0.0010	0.0179	0.0010

Table S3. Comparison of the HER activity of K_2PtCl_4-treated HES obtained in this work with those of representative electrocatalysts reported in the literature.

**Catalysts**	**Electrolyte**	**η_{10} (mV)**	**Tafel slope (mV dec^{-1})**	**Ref.**
(CuAgZnCoMnInGa)S	0.5 M H_2SO_4	255	131.6	25
(CrFeNiCoZn)S	0.5 M H_2SO_4	15	105	23
PdPtCuNiP	0.5 M H_2SO_4	62	44.6	58
Pt/NiFeCoCuCr-LDH	0.5 M H_2SO_4	42	42.0	59
RhRuPtPdIr/GC	0.5 M H_2SO_4	13	14.0	60
$Pd_{20}Pt_{20}Cu_{20}Ni_{20}P_{20}$	0.5 M H_2SO_4	36	34.2	61
$MoS_2/CoNi_2S_4$	0.5 M H_2SO_4	81	75.3	62
Pt-MoS_2	0.5 M H_2SO_4	67.4	76.2	63
CoS/PCE	0.5 M H_2SO_4	190	66.0	64
K_2PtCl_4-treated HES	0.5 M H_2SO_4	49	51.4	This work
$(ZnCoMnFeAlMg)_9S_8$/NF	1.0 M KOH	170	-	65
(CrFeNiCoZn)S	1.0 M KOH	49	235.0	23
$(Fe_{0.2}Mn_{0.2}Ni_{0.2}Co_{0.2}Mo_{0.2})S_2$	1.0 M KOH	187	96.0	24
PdPtCuNiP	1.0 M KOH	32	37.4	58
CoFeLaNiPt	0.1 M KOH	555	-	66
$Co_{0.6}(VMnNiZn)_{0.4}PS_3$	1.0 M KOH	65.9	65.5	53
Rh-Ni_3S_2/Co_3S_4/NF	1.0 M KOH	78	115.2	67
B-Fe_7S_8/FeS_2	1.0 M KOH	113	57.4	68
$MoS_2/CoNi_2S_4$	1.0 M KOH	78	67.4	62
K_2PtCl_4-treated HES	1.0 M KOH	50	55.0	This work

23. V. Buravets, E. Miliutina, V. Burtsev, K. Severa, V. Shilenko, J. Rosenkranzova, P. Hönicke, T. Hrbek, V. Svorcik and O. Lyutakov, *Appl. Surf. Sci*., 2025, **681**, 161600.
24. L. Lin, Z. Ding, G. Karkera, T. Diemant, D. Chen, M. Fichtner, H. Hahn, J. Aghassi-Hagmann, B. Breitung and S. Schweidler, *Mater. Futures*, 2024, **3**, 045102.
25. W. Xiao, Y. Li, A. Elgendy, E. C. Duran, M. A. Buckingham, B. F. Spencer, B. Han, F. Alam, X. Zhong, S. H. Cartmell, R. J. Cernik, A. S. Eggeman, R. A. W. Dryfe and D. J. Lewis, *Chem. Mater*., 2023, **35**, 7904-7914.
53. R. Wang, J. Huang, X. Zhang, J. Han, Z. Zhang, T. Gao, L. Xu, S. Liu, P. Xu and B. Song, *ACS Nano*, 2022, **16**, 3593-3603
58. Z. Jia, K. Nomoto, Q. Wang, C. Kong, L. Sun, L.-C. Zhang, S.-X. Liang, J. Lu and J. J. Kruzic, *Adv. Funct. Mater.*, 2021, **31**, 2101586.
59. H. Xu, Y. Liu, K. Wang, L. Jin, J. Chen, H. Chen and G. He, *J. Colloid Interface Sci.*, 2025, **684**, 566-574.
60. Y. Zou, L. Jing, J. Zhang, S. Luo, L. Wang, Y. Li, R. Goei, K. W. Tan and A. I. Yoong Tok, *J. Mater. Chem. A*, 2024, **12**, 5668-5678.
61. Y. Li, J. Tang, H. Zhang, Y. Wang, B. Lin, J. Qiao, H. Zheng, Z. Yu, Y. Liu, T. Zhou and X. Lei, *Chem. Eng. J.*, 2023, **453**, 139905.
62. J. Hu, C. Zhang, P. Yang, J. Xiao, T. Deng, Z. Liu, B. Huang, M. K. H. Leung and S. Yang, *Adv. Funct. Mater.*, 2020, **30**, 1908520.
63. A. Shan, X. Teng, Y. Zhang, P. Zhang, Y. Xu, C. Liu, H. Li, H. Ye and R. Wang, *Nano Energy*, 2022, 94, 106913.
64. P. M. Pataniya, V. Patel, P. Sahatiya, D. J. Late and C. K. Sumesh, *Surf. Interfaces*, 2022, 34, 102319.
65. S. Li, L. Tong, Z. Peng, B. Zhang and X. Fu, *Green Chem*., 2024, **26**, 384-395.
66. M. W. Glasscott, A. D. Pendergast, S. Goines, A. R. Bishop, A. T. Hoang, C. Renault and J. E. Dick, *Nat. Commun*., 2019, **10**, 2650.
67. J.-C. Gan, Z.-F. Jiang, K.-M. Fang, X.-S. Li, L. Zhang, J.-J. Feng and A.-J. Wang, *J. Colloid Interface Sci.*, 2025, **677**, 221-231.
68. J. Wu, Q. Zhang, K. Shen, R. Zhao, W. Zhong, C. Yang, H. Xiang, X. Li and N. Yang, *Adv. Funct. Mater.*, 2022, **32**, 2107802.

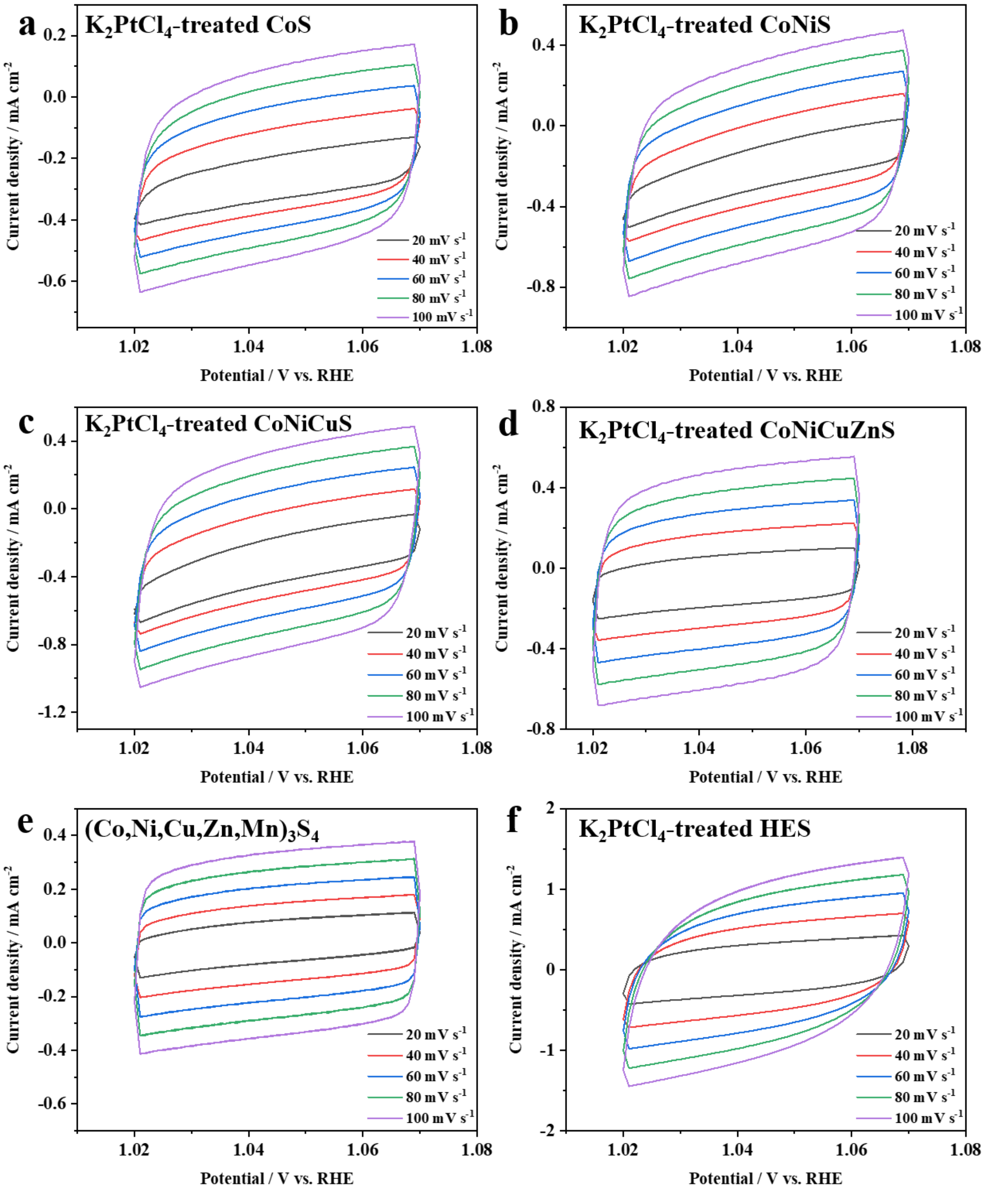

Fig. S9 Cyclic voltammetry curves of $(Co,Ni,Cu,Zn,Mn)_3S_4$, K_2PtCl_4-treated CoS, K_2PtCl_4-treated CoNiS, K_2PtCl_4-treated CoNiCuS, K_2PtCl_4-treated CoNiCuZnS, and K_2PtCl_4-treated HES measured at various scan rates in 1 M KOH.

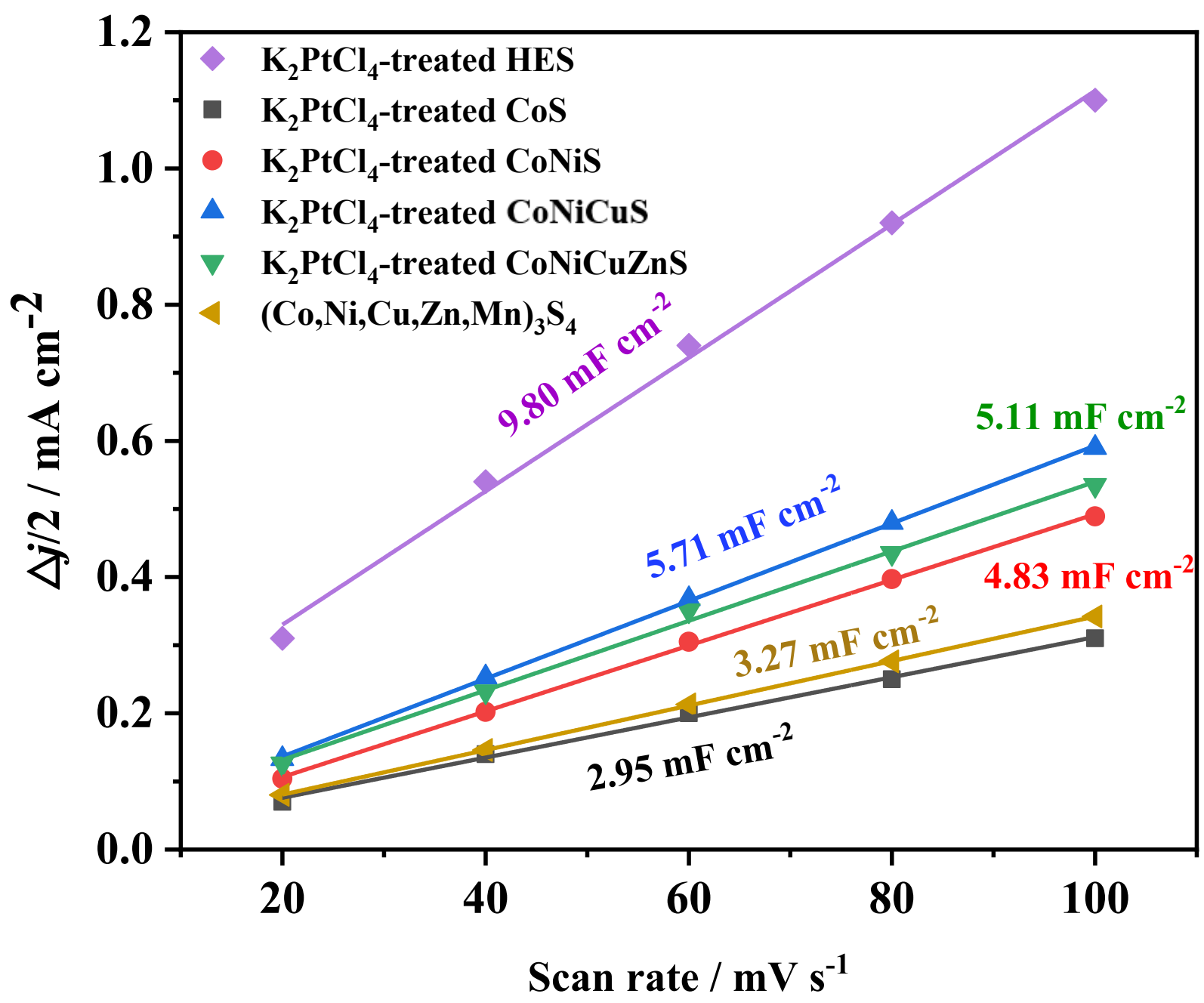

Fig. S10 Electrochemical double-layer capacitance (C_{dl}) of (Co,Ni,Cu,Zn,Mn)$_3$S$_4$, K$_2$PtCl$_4$-treated CoS, K$_2$PtCl$_4$-treated CoNiS, K$_2$PtCl$_4$-treated CoNiCuS, K$_2$PtCl$_4$-treated CoNiCuZnS, and K$_2$PtCl$_4$-treated HES calculated from cyclic voltammetry measurements in 1 M KOH.

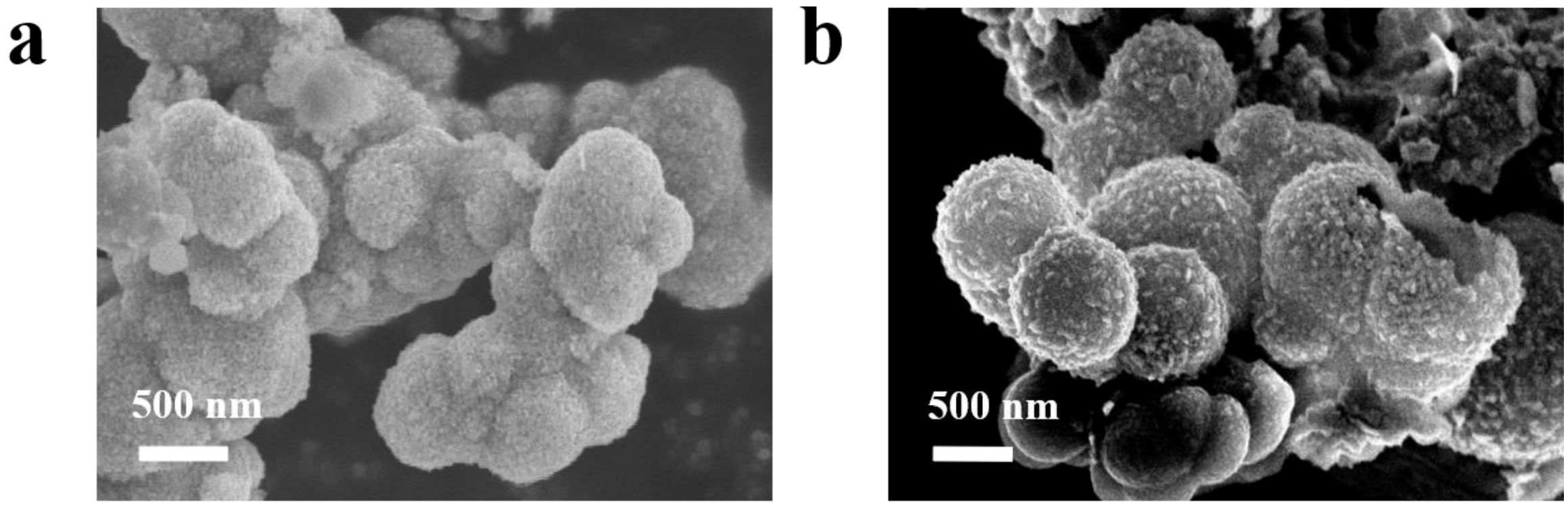

Fig. S11 Comparison of SEM image of K_2PtCl_4-treated HES (with carbon paper) after 60-h *i-t* measurement in (a) 1 M KOH and (b) 0.5 M H_2SO_4.

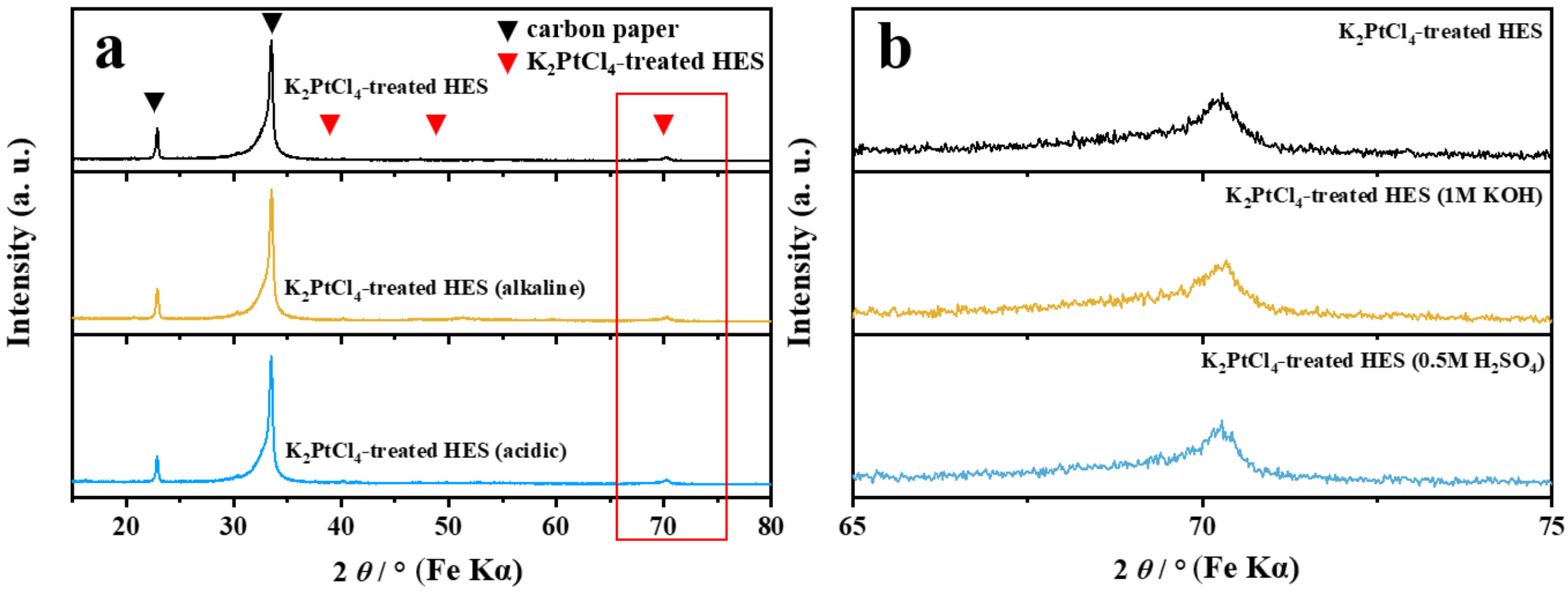

Fig S12 Comparison of XRD pattern of K_2PtCl_4-treated HES (with carbon paper) before and after 60-h *i*-*t* measurements in 1 M KOH and 0.5 M H_2SO_4.

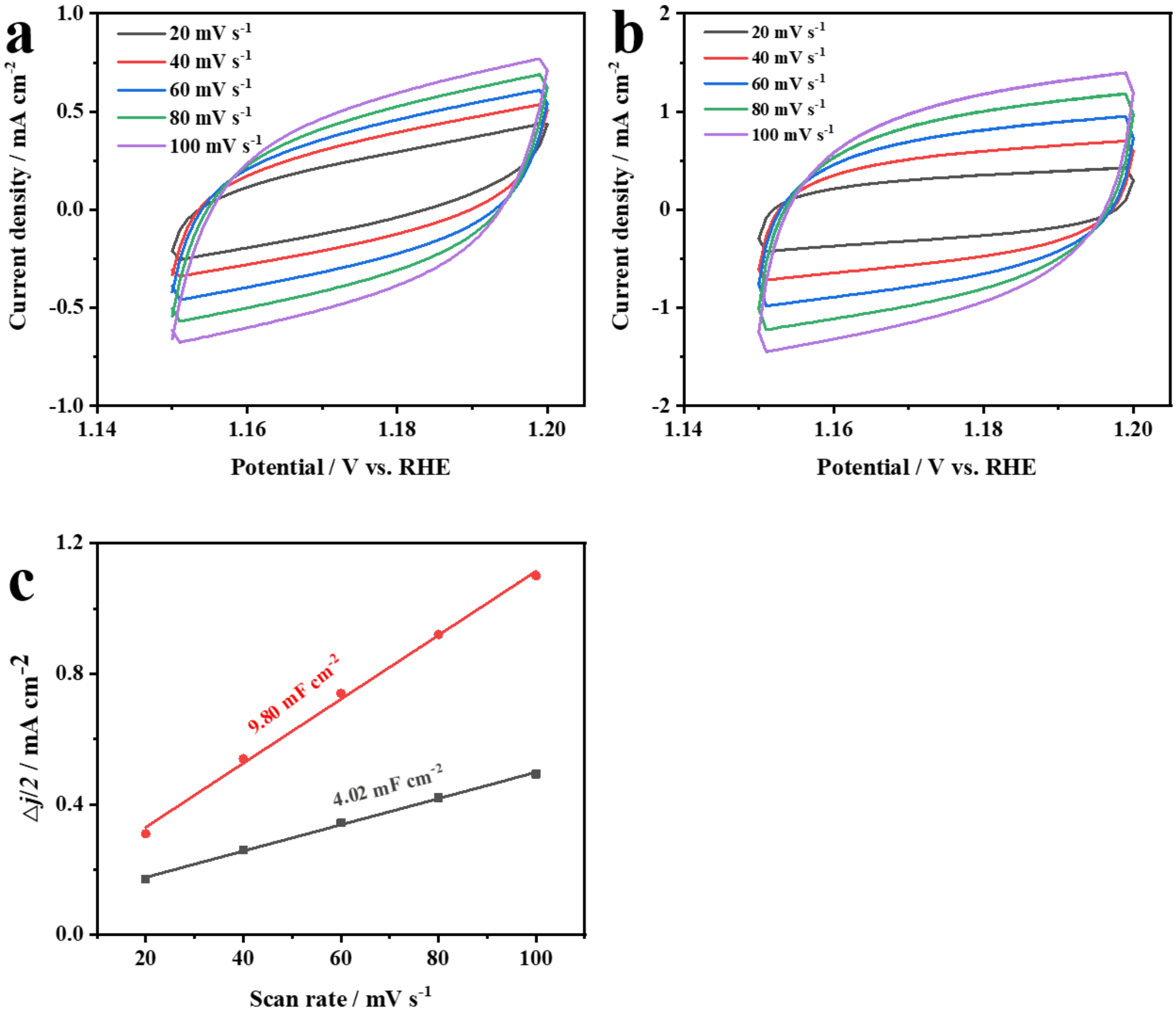

Fig. S13 Cyclic voltammetry curves of K_2PtCl_4-treated HES measured at various scan rates cycles in 1 M KOH (a) before and (b) after 5000 cyclic voltammetry cycles, (c) the corresponding Cdl calculated from cyclic voltammetry curves.

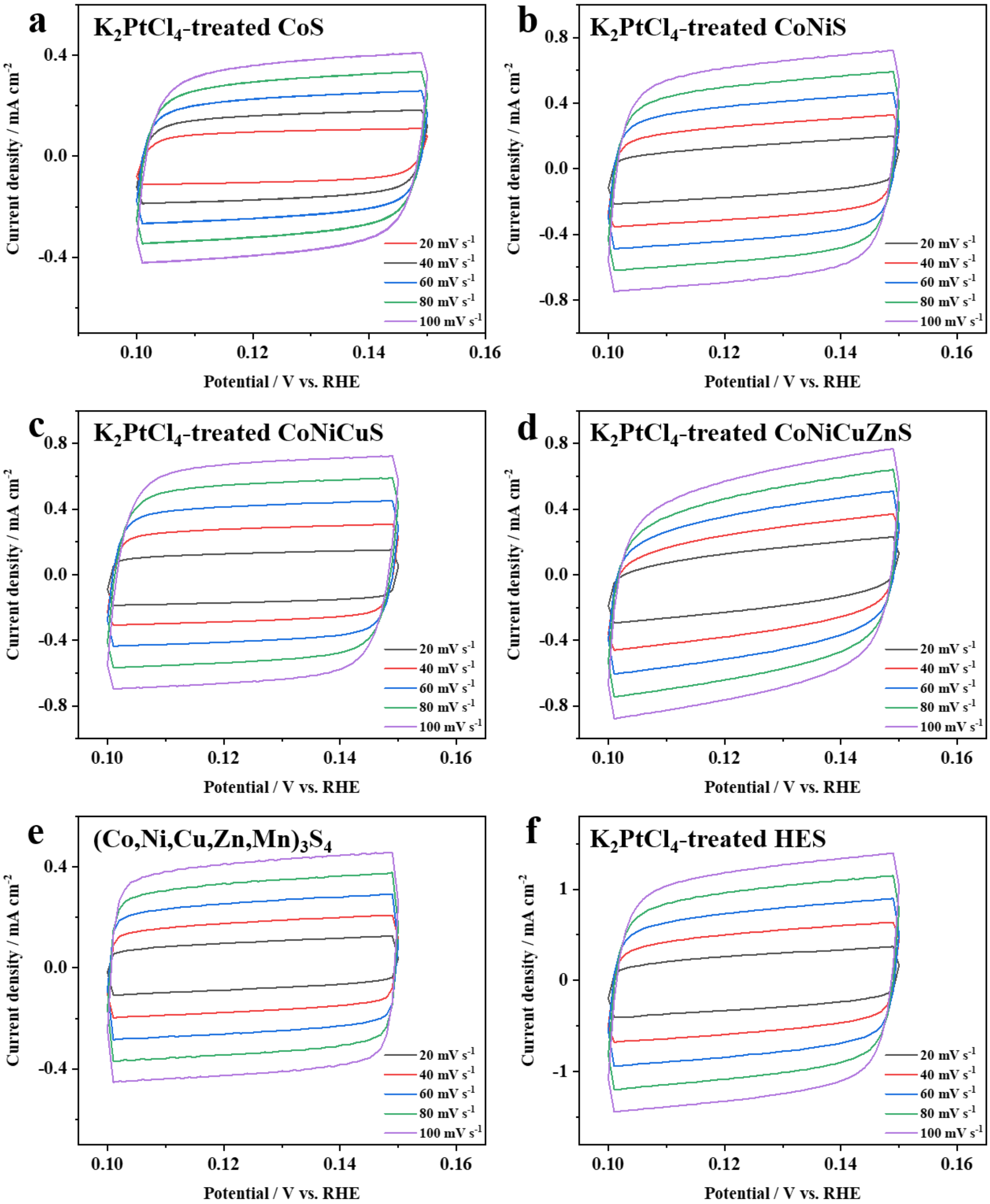

Fig. S14 Cyclic voltammetry curves of $(Co,Ni,Cu,Zn,Mn)_3S_4$, K_2PtCl_4-treated CoS, K_2PtCl_4-treated CoNiS, K_2PtCl_4-treated CoNiCuS, K_2PtCl_4-treated CoNiCuZnS, and K_2PtCl_4-treated HES measured at various scan rates in 0.5 M H_2SO_4.

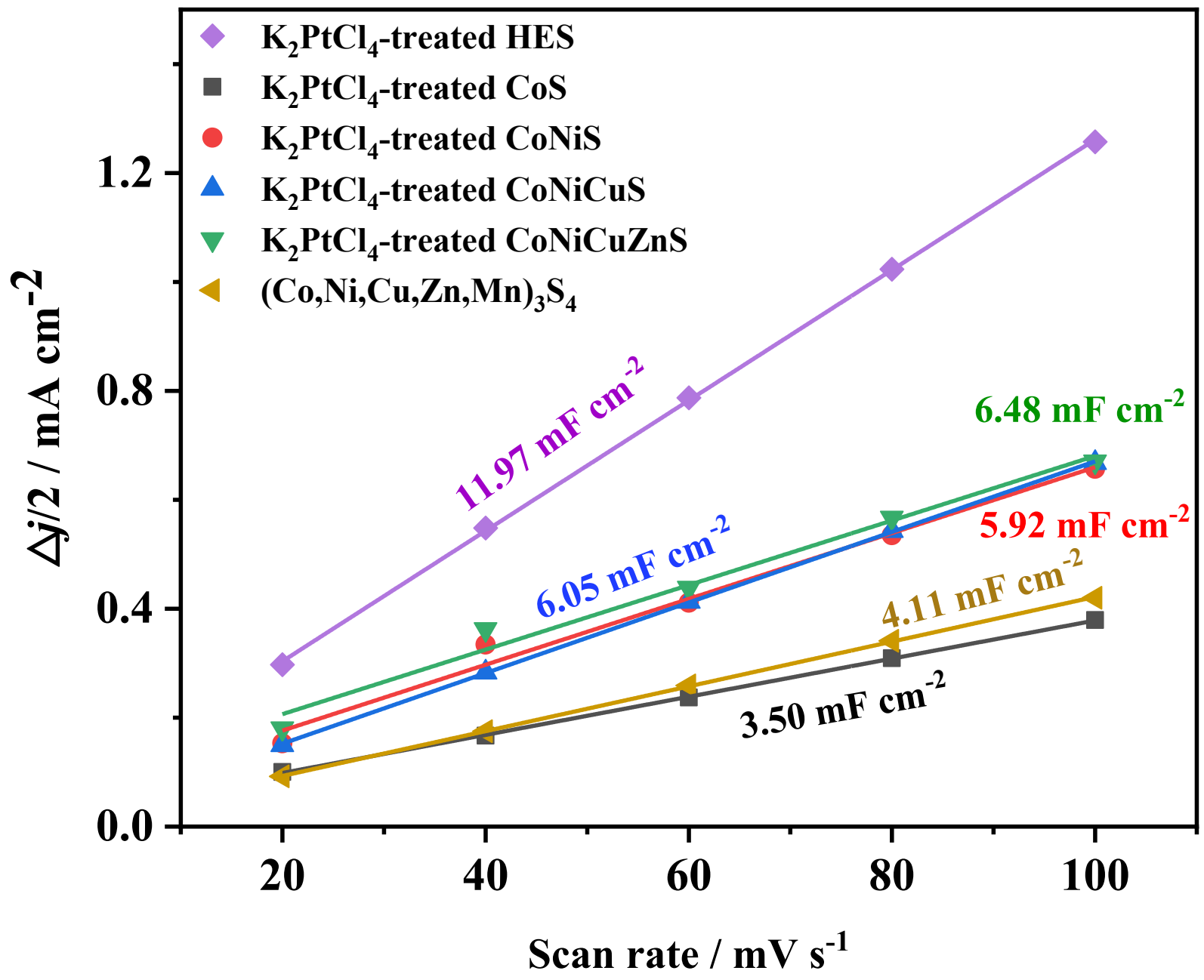

Fig. S15 C_{dl} values of $(Co,Ni,Cu,Zn,Mn)_3S_4$, K_2PtCl_4-treated CoS, K_2PtCl_4-treated CoNiS, K_2PtCl_4-treated CoNiCuS, K_2PtCl_4-treated CoNiCuZnS, and K_2PtCl_4-treated HES calculated from cyclic voltammetry measurements in 0.5 M H_2SO_4.

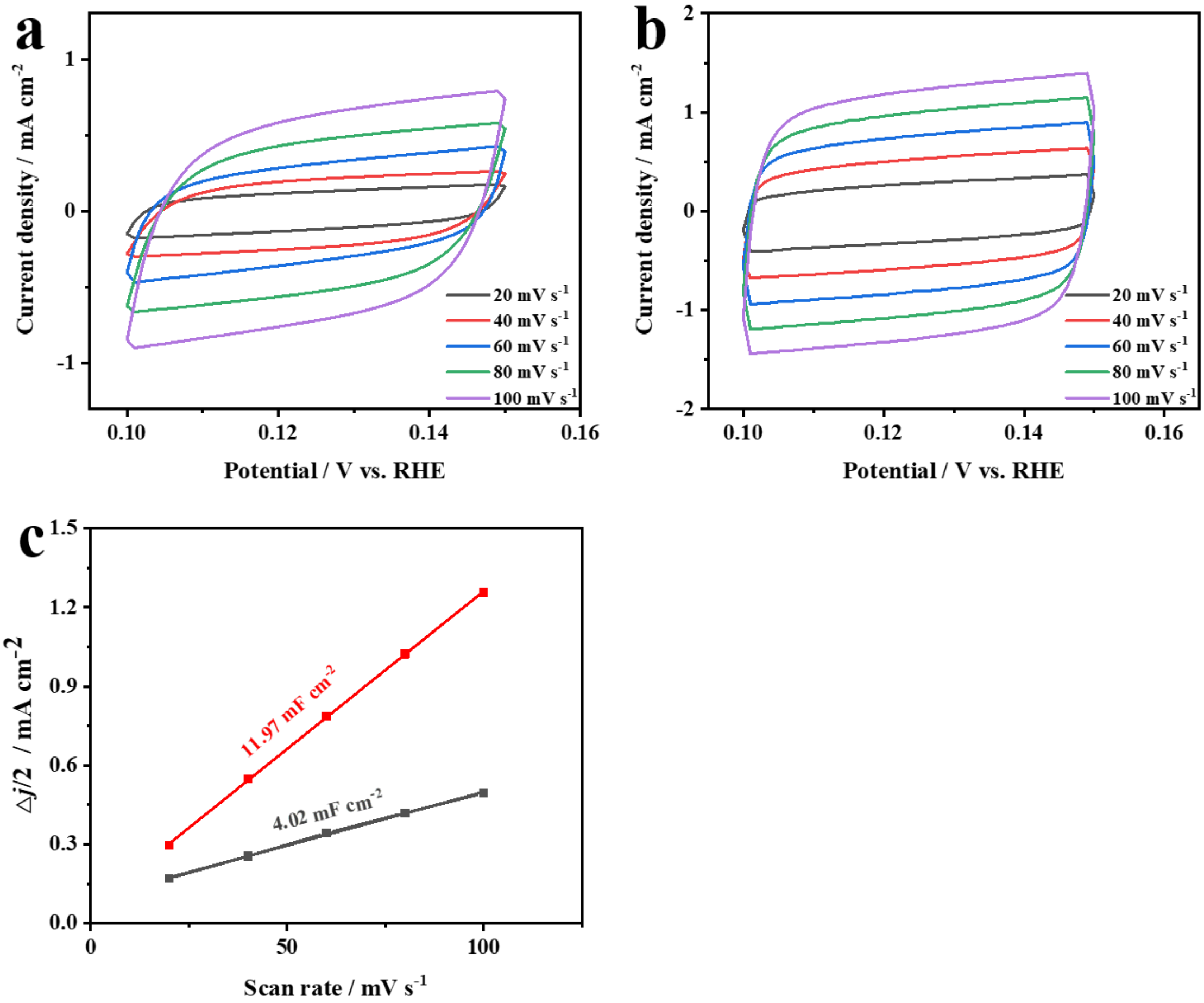

Fig. S16 Cyclic voltammetry curves of K_2PtCl_4-treated HES measured at various scan rates cycles in 0.5 M H_2SO_4 (a) before and (b) after 5000 cyclic voltammetry cycles. (c) The corresponding C_{dl} calculated from cyclic voltammetry curves.
